# Diagnostic yield of panel-based genetic testing in syndromic inherited retinal disease

**DOI:** 10.1038/s41431-019-0548-5

**Published:** 2019-12-13

**Authors:** Omamah A. Jiman, Rachel L. Taylor, Eva Lenassi, Jill Clayton Smith, Sofia Douzgou, Jamie M. Ellingford, Stephanie Barton, Claire Hardcastle, Tracy Fletcher, Christopher Campbell, Jane Ashworth, Susmito Biswas, Simon C. Ramsden, UK Inherited Retinal Disease Consortium, Forbes D. Manson, Graeme C. Black

**Affiliations:** 1 Division of Evolution and Genomic Sciences, School of Biological Sciences, Faculty of Biology, Medicine and Health, Manchester Academic Health Science Centre (MAHSC), University of Manchester, Manchester, UK; 2 Department of Genetic Medicine, Faculty of Medicine, King Abdulaziz University, Jeddah, Saudi Arabia; 3 Manchester Centre for Genomic Medicine, St Mary’s Hospital, Central Manchester University Hospitals NHS Foundation Trust, MAHSC, Manchester, UK; 4 Manchester Royal Eye Hospital, Central Manchester University Hospitals NHS Foundation Trust, Manchester Academic Health Science Centre, Manchester, UK

**Keywords:** Genetic testing, Disease genetics, Next-generation sequencing, Medical genetics

## Abstract

Thirty percent of all inherited retinal disease (IRD) is accounted for by conditions with extra-ocular features. This study aimed to establish the genetic diagnostic pick-up rate for IRD patients with one or more extra-ocular features undergoing panel-based screening in a clinical setting. One hundred and six participants, tested on a gene panel which contained both isolated and syndromic IRD genes, were retrospectively ascertained from the Manchester Genomic Diagnostics Laboratory database spanning 6 years (2012–2017). Phenotypic features were extracted from the clinical notes and classified according to Human Phenotype Ontology; all identified genetic variants were interpreted in accordance to the American College of Medical Genetics and Genomics guidelines. Overall, 49% (*n* = 52) of patients received a probable genetic diagnosis. A further 6% (*n* = 6) had a single disease-associated variant in an autosomal recessive disease-relevant gene. Fifty-two percent (*n* = 55) of patients had a clinical diagnosis at the time of testing. Of these, 71% (*n* = 39) received a probable genetic diagnosis. By contrast, for those without a provisional clinical diagnosis (*n* = 51), only 25% (*n* = 13) received a probable genetic diagnosis. The clinical diagnosis of Usher (*n* = 33) and Bardet–Biedl syndrome (*n* = 10) was confirmed in 67% (*n* = 22) and 80% (*n* = 8), respectively. The testing diagnostic rate in patients with clinically diagnosed multisystemic IRD conditions was significantly higher than those without one (71% versus 25%; *p* value < 0.001). The lower pick-up rate in patients without a clinical diagnosis suggests that panel-based approaches are unlikely to be the most effective means of achieving a molecular diagnosis for this group. Here, we suggest that genome-wide approaches (whole exome or genome) are more appropriate.

## Introduction

Inherited retinal disease (IRD) refers to a group of clinically and genetically heterogeneous conditions affecting the retina [1]. IRD is usually confined to the eye but up to 30% of affected individuals are considered syndromic as they have one or more associated extra-ocular features [2], some of which require health surveillance or specific clinical management measures to be put in place. Making a timely definitive diagnosis in IRD cases complicated by extra-ocular features is important, therefore, but is often challenging [3, 4]. More than 260 genes have been associated with IRD; over 100 of these have been linked to conditions of IRD with extra-ocular features. These disorders are associated with autosomal dominant (AD), autosomal recessive (AR) and X-linked inheritance (RetNet, http://www.sph.uth.tmc.edu/RetNet/).

There are a small number of well known, relatively prevalent multisystemic conditions that feature IRD as a prominent feature; including Bardet–Biedl syndrome (BBS) and Usher syndrome. The diagnostic pick-up rate of molecular genetic testing for these syndromes is high as the majority of disease-associated genes have been discovered. Variants in at least 24 genes are found in ~80% of individuals affected with BBS, a ciliopathy that impacts multiple body systems including the eye and the kidney [5, 6]. Usher syndrome, a ciliopathy manifesting with IRD and sensorineural hearing impairment (SNHI), is caused by variants in 15 genes (RetNet). Two other such conditions where a clinical diagnosis can be made and chances of finding a diagnosis is high are Cohen syndrome (*VPS13B*) and Alström syndrome (*ALMS1*). Genes related to these conditions are sometimes included in an IRD NGS testing approach.

Genomic testing provides a fast, cost-effective and reliable way of interrogating multiple genes at one time [7]. Over the past decade, this technology has revolutionised the diagnosis of genetically heterogeneous conditions like IRD [8]. Importantly, there is a lack of studies that have examined the yield of genetic testing in syndromic IRD in general as most reports focus on either isolated IRD, or on specific conditions such as Usher syndrome [7–12]. A panel-based NGS test for IRD genes was designed by the Genomic Diagnostics Laboratory within Manchester Centre for Genomic Medicine (MCGM) in 2012 to genetically test for a custom group of genes known to be causative for both isolated and syndromic IRD conditions. Our hypothesis was that cases with distinctive multisystemic presentations would more likely benefit from this genetic testing strategy compared with those in which such a diagnosis had not been formulated clinically. Aiming to gain further insight into this, we have assessed the clinical utility of our panel-based genetic testing approach in individuals with assumed syndromic IRD. In addition, we have described the range of genes and genomic variants in this cohort and compared them with the literature.

## Subjects and methods

### Recruitment and phenotype data collection

The cohort was assembled retrospectively from a database of patients referred for genetic testing to the Genomic Diagnostics Laboratory within MCGM; (Manchester, UK) between January 2012 and December 2017. Before genetic testing, all study participants were examined by a clinical geneticist and a paediatric and/or genetic ophthalmologist within the Manchester University NHS Foundation Trust (Manchester, UK), and then subsequently referred for genetic testing at the Genomic Diagnostics Laboratory at MCGM. Only patients referred with an IRD with at least one non-ocular feature were included in this study. For the purpose of this study, an individual is considered to have a syndromic IRD if they have ophthalmic signs and symptoms suggestive of retinal disease (including those with inherited retinal dystrophies and retinal dysplasia) and at least one feature identified by a clinical geneticist to be potentially an associated manifestation of a multisystem disorder. This might be, for example, a structural malformation, hearing loss, or a significant growth or developmental disorder. Informed consent was obtained from all patients prior to panel-based clinical genetic testing and the study protocol observed the tenets of the Declaration of Helsinki.

The clinical notes were reviewed and all relevant phenotypic features were collected and converted into Human Phenotype Ontology (HPO) terms using the HPO Browser (http://compbio.charite.de/hpoweb/ (accessed between February and July 2018) [13]. The clinical diagnosis of a specific syndromic condition was provisionally made when the phenotype fulfilled predefined diagnostic criteria (Supplementary Table S1) or when there was a high index of suspicion for a specific diagnosis. We used the diagnostic criteria set by Beales et al. (1999) to define a clinical diagnosis of BBS [14] (Supplementary Table S1). Also, for Senior–Loken syndrome (SLS), a high index of suspicion was recorded in an individual with apparently isolated IRD because a family history of SLS was suspected.

### Genetic testing and bioinformatics analysis

IRD panel testing and data analysis were performed at the Genomic Diagnostics Laboratory within MCGM (Clinical Pathology Accredited no. 4015). Sequencing and bioinformatics analyses have been described previously [15, 16]. Briefly, panel tests were custom designed to include the coding regions (±50 bp of flanking intronic sequence) of either 105 genes (41 samples tested between January 2012 and June 2014) or 176 genes (65 samples tested between July 2014 and December 2017) that have previously been associated with isolated and/or syndromic IRD; lists of the genes included in each panel can be found in Supplementary Tables S2 and S3. Notably, the 105 gene panel included 14 BBS-associated genes and 8 Usher syndrome associated genes, while the 176 gene panel included 22 BBS-associated genes and 11 Usher syndrome associated genes.

Samples were processed using the Agilent SureSelectXT (Agilent Technologies, Santa Clara, USA) target enrichment chemistry, and sequenced on an Illumina HiSeq 2000/2500 or NextSeq500 system (Illumina Inc, San Diego, USA) according to the manufacturers’ protocols. Sequence reads were de-multiplexed using CASAVA software version 1.8.2 (Illumina) and aligned using the Burrows Wheeler Aligner (BWA-short version 0.62) to the hg19 reference genome [17]. Duplicate reads were removed and the Genome Analysis Tool Kit (GATK-lite version 2.0.39) was used for single-nucleotide variant and insertion–deletion discovery [18]. Copy number variants (CNVs) were detected from high-throughput sequencing read data using ExomeDepth version 1.1.6 as described in references [19, 20].

### Variant validation and classification

Clinical interpretation of genetic variants was performed by UK National Healthcare Service (NHS) clinical scientists within the Genomic Diagnostics Laboratory at MCGM. Only variants with a minor allele frequency of <1% in Exome Variant Server (NHLBI GO Exome Sequencing Project (ESP), Seattle, WA were considered. Release ESP6500SI-V2. http://evs.gs.washington.edu/EVS/) and dbSNP were considered. Variant interpretation was supported by in silico pathogenicity predictions (i.e. from SIFT, PolyPhen2 and AlignGVGD), extensive evaluation of the scientific literature, and the patients’ clinical referral, in line with the American College of Medical Genetics and Genomics best practice guidelines [21]. Segregation studies were also performed where clinically appropriate, when relevant samples were available for analysis. For the purpose of this study, molecular genetic diagnoses were classified into three groups using a previously described strategy [16]: (1) Probable genetic diagnosis: a clearly or likely disease-associated variant(s) identified in a gene relevant to the patient’s phenotype, which is present in an apparently disease-causing state (i.e. biallelic variants in AR disease-associated genes and monoallelic variants in AD disease-associated genes); (2) possible genetic diagnosis: a single clearly or likely disease-associated variant identified in a recessive disease gene that is known to cause a spectrum of phenotypic features that match the patients clinical presentation; it may be reasoned that the patient harbours a second change that could not be detected by the chemistry/technology of the test used for diagnosis; (3) unknown genetic diagnosis: no likely or causal disease-associated variant(s) detected. Variants that were concluded likely to contribute to a patients’ molecular diagnosis were confirmed by an alternative method (i.e. Sanger sequencing) and have been submitted to the ClinVar database [https://www.ncbi.nlm.nih.gov/clinvar/] [22] under the searchable submission name: (SyndromicIRD_variants), if not already done so during our previous work (ClinVar accession numbers: SCV000259090.1, SCV000259091.1, SCV000259087.1, SCV000259094.1, SCV000259095.1, SCV000259101.1, SCV000259102.1, SCV000282652.1, SCV000282640.1, SCV000259083.1, SCV000259082.1, SCV000259084.1, SCV000259085.1, SCV000493124.1, SCV000493116.1, SCV000493114.1) (REF: [15, 19, 23]). On occasions where more than one ‘probable’ or ‘possible’ disease-associated variant was identified in disease-causing state, family history, the patients phenotypic presentation, and evidence of variant pathogenicity were considered in detail by a multidisciplinary team. Although all variants are detailed in clinical reports issued by the diagnostic laboratory, only the variant(s) considered most likely to be causal (and/or that had been confirmed to segregate with disease within a family) was included for the purpose of our analysis. It is also of note that ‘probable’ or possible’ disease-associated variants identified in carrier state were clinically reported as carrier findings.

### Statistical analysis

A comparison of the genetic pick-up rate between the two groups was performed using chi-squared test of independence to determine the dependence of a genetic diagnosis by the panel test on having a provisional clinical diagnosis, the statistical significance was defined as *α* = 0.001.

## Results

**Fig. 1:**
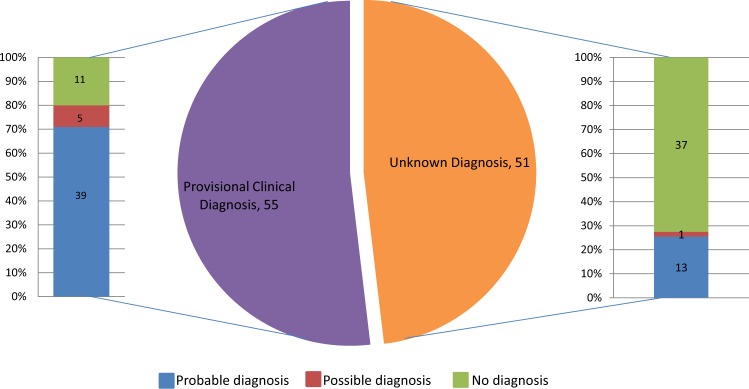
Diagnostic pick-up rate by IRD NGS panel testing in patients with potential syndromic IRD. Of the 55/106 (52%) patients who were referred with a provisional clinical diagnosis, 71% received a probable genetic diagnosis compared with 25% of those patients with no known clinical diagnosis (51/106).

A total of 106 patients were included in our analysis, 61 females and 45 males. Age at referral ranged between 0 days and 69.5 years with a median age of 21.9 years; 43% of study participants were 16 years or younger. Patients fell into one of the two categories (Fig. 1):

(i) Fifty-two percent (55/106) had a provisional clinical diagnosis of a specific syndromic condition (e.g. BBS or Usher syndrome) at the time of referral for genetic testing or,
(ii) forty-eight percent (51/106) referred as probably syndromic IRD, with no clinically recognised diagnosis.

There was a wide range of extra-ocular phenotypes; the most common included hearing impairment (any type/severity), developmental delay (DD)/intellectual disability (ID) (any type, specific/global), polydactyly, microcephaly, obesity, abnormal facial shape (dysmorphic) and kidney/renal abnormalities; a list of HPO terms generated from the clinical notes of the study population can be found in Supplementary Table S4.

A probable genetic diagnosis was made in 49% (52/106) of cases; 6% (6/106) of patients received a possible genetic diagnosis (i.e. they were found to be heterozygous for a variant in an AR gene relevant to the patient’s phenotype). The test failed to detect any likely disease-associated variants in 45% (48/106). Notably, of the 55 patients referred with a specific clinical diagnosis, 39 (71%) received a probable, and confirmed, genetic diagnosis, and 5 (9%) a possible genetic diagnosis. For the 51 patients referred without a specific clinical diagnosis, 13 (25%) received a probable diagnosis and 1 patient received a possible genetic diagnosis (2%) (Fig. 1). The difference was significant, *χ*^2^ (2, *N* = 106) = 31.8264, *p* < 0.001.

### Referrals with a provisional clinical diagnosis

#### Usher syndrome

**Fig. 2:**
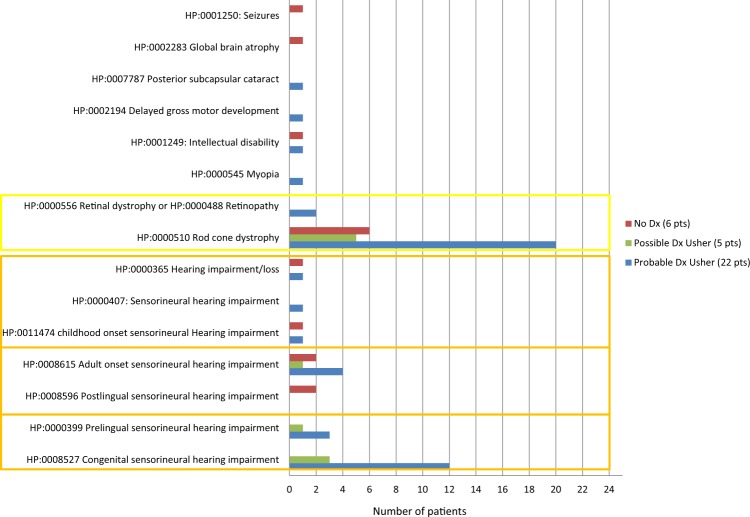
Clinical features in HPO terms found in 33 patients referred with a provisional clinical diagnosis of Usher syndrome. Features in the orange and yellow boxes are the two main features of Usher syndrome that were used as the diagnostic criteria for Usher syndrome in this study. Types and age of onset of hearing impairment in the patients are shown as well as the type of retinal dystrophy and any unusual features found in these patients. The patients are divided by the genetic diagnosis received from the IRD NGS panel test: probable-, possible- and no genetic diagnosis.

Thirty-three patients, 25 (76%) females and 8 (24%) males, aged between 5 months and 58.5 years (median age of 29.3 years), had a provisional clinical diagnosis of Usher syndrome (Supplementary Table S5 and Supplementary Fig. S1). Additional atypical syndromic features were identified in three of these Usher syndrome patients: one patient who was found to have biallelic variants in *PCDH15* had ID, two patients who did not receive a genetic diagnosis: one had global brain atrophy with ID and the other had seizures and migraines (Fig. 2).

Panel testing enabled a probable genetic diagnosis to be identified in 67 % (22/33) of individuals confirming the clinical diagnosis of Usher syndrome. A further 15% (5/33) received a possible genetic diagnosis and no causal variants were found in the remaining 18% (6/33) of patients with clinical Usher syndrome.

The diagnostic pick-up rate differed according to the age of onset of SNHI; post-lingual SNHI was associated with lower yield of genetic testing (Orange boxes in Fig. 2). Regarding family history, ten patients had a family history of a similar condition. Five had congenital/pre-lingual SNHI of which four received a probable genetic diagnosis and one a possible diagnosis. Three had post-lingual SNHI of which two received a probable diagnosis and one a possible diagnosis. Lastly, two patients had unknown ages of onset of SNHI and both received a probable genetic diagnosis (Supplementary Fig. S1).

Two patients with congenital SNHI (one with a family history of Usher and one without) who were found to have a single variant in a gene known to be mutated in Usher syndrome (possible genetic diagnosis), as well as one of the six individuals with no genetic diagnosis, were subject to further testing using whole genome sequencing (WGS). A second heterozygous disease-associated variant was identified in both patients (patients #25 and #26) with a possible genetic diagnosis, confirming the clinical diagnosis of Usher syndrome. In the one patient with no genetic diagnosis following testing of the 105 gene panel (patient #31), subsequent WGS revealed a homozygous deletion of exons 1–7 of *MERTK* gene (NM_006343.2:c.[(?_-1)_(1144+1_1145-1)del];[(?_-1)_(1144+1_1145-1)del]). This deletion has been previously identified in heterozygous state in another in-house patient with isolated rod-cone dystrophy [23]. A 91 kb deletion including this region is a common founder variant in the Faroe Islands, associated with non-syndromic AR rod-cone dystrophy without hearing impairment [24]. No variants in genes associated with hearing impairment were identified in this individual who was diagnosed with hearing impairment in his late teenage years.

Overall, 42 different disease-associated variants were found in 6 known Usher syndrome genes. Of these, 14 have not previously been associated with Usher syndrome. The genes involved and variant types are shown in Supplementary Fig. S2.

#### Bardet–Biedl Syndrome

**Fig. 3:**
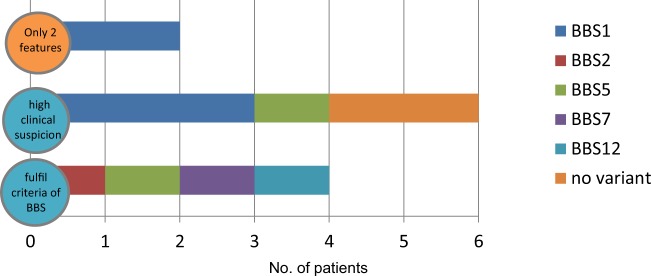
Phenotype–genotype correlation in BBS. BBS was diagnosed in 8/10 patients who fulfilled the diagnostic criteria or with high index of suspicion. In addition, the NGS panel was able to pick-up two early cases with only two features. *BBS1* was causative in half of the diagnosed cases.

Ten patients were referred with a high index of suspicion for BBS (Fig. 3). Four were male and six female, and age at referral ranged between 3 and 41 years (median was 16.8 years) (Supplementary Table S6). Eight of these ten patients received a probable genetic diagnosis of BBS after panel testing; no genetic diagnosis was made in the remaining two study subjects. Seven of the eight patients with a probable genetic diagnosis were homozygous for the disease-causing variant and one patient harboured a compound heterozygous variant.

Interestingly, the panel test revealed a probable genetic diagnosis of BBS in two additional patients who were in the unknown clinical diagnosis group, reflecting the observation that the clinical spectrum of BBS is now recognised to be wide. The mutated genes and variant types found in all ten patients with a probable genetic diagnosis of BBS are shown in Supplementary Fig. S3.

#### Other IRD multisystemic conditions

One patient was referred with a clinical diagnosis of Joubert syndrome (Supplementary Table S7). Genetic testing revealed a previously reported homozygous nonsense variant in *CEP290.* NM_025114.3:c.5668G>T; p.(Gly1890*) confirming the diagnosis [25, 26].

Five patients, two male and three female, were referred with, or fulfilled the diagnostic criteria for, SLS. Median age at referral was 28.9 years (range 9.3–58.8 years). All five patients had rod-cone dystrophy and of the four study subjects who had renal insufficiency; nephronophthisis (a key indicator of SLS) was specifically noted in only one patient (Supplementary Table S8). One 29-year-old patient (#46 in Supplementary Table S8) did not have renal impairment but due to a family history of SLS, she was highly suspected to have SLS. Panel-based testing identified a probable molecular diagnosis in four of these five patients (80%) with presumed SLS, including patient #46. Six different disease-associated variants were identified in three different SLS genes (*CEP290, IQCB1* and *NPHP4*).

Five patients were referred with Cohen syndrome; three male and two female. Age at referral ranged between 3.3 years and 49.2 years with a median age of 18 years. All five patients fulfilled previously outlined diagnostic criteria [27] (Supplementary Table S9). A probable genetic diagnosis was made in three of five patients (60%); no disease-associated variants or CNVs were identified in the remaining two individuals. Each genetically diagnosed patient had different compound heterozygous variants in *VPS13B*, resulting in six different variants in total, one of which has not been previously reported. The three genetically confirmed Cohen syndrome patients had four features in common: characteristic facial dysmorphism, microcephaly, a form of DD, and an ocular phenotype.

One male patient was referred with Norrie disease at the age of 13 months (Supplementary Table S10). The panel test found a heterozygous missense variant NM_012193.3:c.313A>G, p.(Met105Val) in *FZD4*, which has been associated with AD familial exudative vitreoretinopathy [28, 29].

### Referrals with unknown diagnoses

Fifty-one patients, 27 male and 24 female, with syndromic IRD were referred for IRD panel testing with no specific clinical diagnosis at the time of referral. Ages at referral ranged between 0 days and 69.5 years (median 14.2 years). Extra-ocular manifestations were highly variable, the most common being DD (global or specific), ID, hearing impairment and obesity. Twenty-five patients had one extra-ocular feature, 15 patients had two extra-ocular features, and 11 patients had 3–6 extra-ocular features (Supplementary Table S11).

Panel testing identified a probable genetic diagnosis in 13 of these 51 patients (25%) and a possible genetic diagnosis in 1 of these 51 (2%) patients; the remaining 37 of 51 patients (73%) received no genetic diagnosis. As mentioned, two patients who had not been suggested to have BBS—due to only having two primary features (one had rod-cone dystrophy and ID and the other had rod-cone dystrophy with polydactyly)—were in fact found to have homozygous variants in *BBS1*. Five patients received a probable genetic diagnosis due to the identification of likely disease-associated variants underlying one of the following syndromes: two patients with homozygous or compound heterozygous variants in *MVK* which is associated with the AR conditions mevalonic aciduria and hyper-IgD syndrome; one with a heterozygous change in *OTX2* which is associated with AD syndromic microphthalmia type 5; one with a homozygous change in *IFT140* which is associated with AR short-rib thoracic dysplasia 9 (with or without polydactyly); and one with a heterozygous change in *KIF11* which is associated with AD microcephaly (with or without chorioretinopathy, lymphedema or mental retardation). Interestingly, six patients had probable disease-associated variants that only explained their retinal phenotype but not their extra-ocular manifestations (patients 64–69 in Supplementary Table S11; variants in the *GNAT2, CNGA3, PROM1, PRPF3, EYS* and *USH2A* genes).

Overall, in this unknown diagnosis group, panel testing identified 17 different disease-causing variants in 12 genes. Four of the variants are previously unreported and a further five have been exclusively reported by our group (Table 1) [16].

**Table 1:** Seventeen variants found in fourteen patients with unknown clinical diagnosis.

Variant	Type of variant	Patient phenotype	Publications if published
MVK NM_000431.2:c.380C>T p.(Pro127Leu) hom	Missense	HP:0001263 global developmental delay; HP:0000365 hearing impairment/loss; HP:0000821 hypothyroidism; HP:0001508 failure to thrive; HP:0001410 decreased liver function; HP:0000548 cone/cone-rod dystrophy	Published by our group (same patient): [16]
MVK NM_000431.2:c.630G>A p.(Trp210Ter) het,	Nonsense	HP:0010978 abnormality of immune system physiology; HP:0000510 retinitis pigmentosa;	Novel
MVK c.1129G>A p.(Val377Ile) het	Missense		[38, 39]
KIF11 NM_004523.3:c.478_479delCT p.(Leu160ValfsTer5) het	Frameshift deletion	HP:0000252 microcephaly; HP:0011968 feeding difficulties; HP:0001263 global developmental delay; HP:0007973 retinal dysplasia; HP:0007773 vitreoretinopathy	Published by our group (same patient): [16]
BBS1 NM_024649.4:c.1110G>A p.(Pro370Pro) Hom.	Synonymous	HP:0000662 nyctalopia; HP:0000510: rod-cone dystrophy; HP:0001249 intellectual disability	[40]
BBS1 NM_024649.4:c.1169T>G p.(Met390Arg) Hom	Missense	HP:0000510 rod-cone dystrophy; HP:0010442 polydactyly; HP:0002099 asthma; HP:0100502 Vitamin B12 deficiency	[41, 42]
OTX2 NM_021728.2:c.811delA p.(Thr271LeufsTer31) het	Frameshift deletion	HP:0001250 seizures; HP:0000545 myopia; HP:0000510 retinitis pigmentosa; HP:0000662 nyctalopia; HP:0007663 reduced visual acuity; HP:0000518 cataract; HP:0000639 nystagmus	Novel
IFT140 NM_014714.3:c.998G>A p.(Cys333Tyr) homozygous	Missense	HP:0001251 cerebellar ataxia;HP:0000510 retinitis pigmentosa	[43, 44]
Genes associated with isolated retinal dystrophy			
GNAT2 NM_005272.3:c.605G>A p.(Gly202Glu) hom	Missense	HP:0000750 delayed speech and language development; HP:0000548 cone/cone-rod dystrophy	Published by our group (same patient): [16]
CNGA3 NM_001298.2:c.560T>C p.(Ile187Thr) hom.	Missense	HP:0001249 intellectual disability; HP:0000548 cone/cone-rod dystrophy	Published by our group (same patient): [16]
TRPM1 NM_002420.5:c.2951G>A p.(Arg984His) Het;	Missense	HP:0001249 intellectual disability;HP:0000708 behavioural abnormality;HP:0007642 congenital stationary night blindness	[45]
microarray identified a 15q13.3 microdeletion reported in the loss of the TRPM1 gene: NM_002420.5:c.(?_−1)_(*1_?)del	Microdeletion		[19]
PROM1 NM_006017.2:c.1354dupT p.(Tyr452Leufs*13) hom	Frameshift duplication	HP:0001251 ataxia;HP:0000556 retinal dystrophy	[46, 47]
PRPF3 NM_004698.2:c.1285G>T p.(Asp429Tyr) het	Missense	HP:0002652 skeletal dysplasia;HP:0000510 retinitis pigmentosa	Novel
EYS NM_001142800.1:c.8133_8137del p.(Phe2712CysfsTer33) het	Frameshift deletion	HP:0001513 obesity;HP:0000510 retinitis pigmentosa;	[48]
EYS NM_001142800.1:c.8816G>C p.(Cys2939Ser) het	Missense		Novel
USH2A c.6670G>T p.(Gly2224Cys) het	Missense	HP:0001249 intellectual disability; HP:0000510 retinitis pigmentosa	[49]
USH2A NM_206933.2:c.10342G>A p.(Glu3448Lys) het	Missense		[50]

*Hom* homozygous, *com. Het.* compound heterozygous

In one patient with congenital stationary night blindness and ID and a possible genetic diagnosis by panel testing, heterozygous missense variant NM_002420.5:c.2951G>A; p.(Arg984His) in *TRPM1*, previous array comparative genomic hybridisation screening identified a heterozygous 15q13.3 microdeletion in the trans allele which resulted in loss of the *TRPM1* gene NM_002420.5:c.(?_−1)_(*1_?)del.

## Discussion

The aim of this study was to determine the efficacy of providing a genetic diagnosis using panel-based testing in individuals with IRD and at least one potentially associated non-ocular feature. Although the panel-based strategy had proven useful in isolated ocular disease, we were more circumspect about whether this was the best way to investigate syndromic retinal disorders and in particular we hypothesised that cases with phenotypes typical of specific multisystemic conditions would have a higher rate of genetic diagnosis compared with those with phenotypes not aligning with any such condition. We also aimed to describe the range of genes and variants in this cohort and to compare these with other studies. A diverse population of 106 patients, of predominantly European origin, were included in our analysis. Our study included 55 cases with a provisional clinical diagnosis of a known condition and 51 cases without a clinical diagnosis of a specific condition at the time of testing. The diagnostic pick-up rate of genetic testing (probable genetic diagnosis) was significantly higher (71%) in the former when compared with the latter group (25%) (*p* < 0.001).

We identified biallelic or monoallelic variants in 67% and 15% of clinically diagnosed Usher syndrome patients, respectively. This is lower compared with the 93 and 6% pick-up rate quoted in a large cohort of 427 patients recruited in various European medical centres [30]. For BBS, we identified biallelic variants in eight of the ten cases referred with a provisional clinical diagnosis of BBS; this is similar to the frequently quoted figure of 80% yield of genetic testing in individuals with clinically diagnosed BBS [5, 31]. Given that the genetic architecture of these syndromes has, to a great extent, been deciphered we believe that any differences in diagnostic pick-up rate are most likely due to patient ascertainment factors, genetic test factors (quality and comprehensiveness of the test) or population structure factors.

We use Usher syndrome as an example: when we focused our analysis on individuals with Usher syndrome and pre-lingual SNHI (i.e. excluding mild/equivocal cases), we found that all 18 study subjects received a possible or probable genetic diagnosis; in contrast, the diagnostic yield in patients with presumed Usher syndrome and post-lingual SNHI was four out of nine patients (Fig. 2). It could also be noteworthy that regardless of Usher classification, family history of disease appears to have no bearing on the likelihood of a probable genetic diagnosis from panel-based testing (Supplementary Fig. S1). However, due to the relatively low numbers within each classification in our current cohort, it is not possible to draw a definitive conclusion at this time. Although there is an apparent difference in diagnostic yield between this study and the report by Bonnet et al., the latter employed a different testing strategy that included looking for possible large genomic rearrangements by genome-wide single-nucleotide variant array analysis, an approach that increased the final yield in that study by 9% [30]. In addition, their patient cohort consisted of only 2% with post-lingual SNHI compared with our 27%. Finally, a large study of 119 Usher syndrome patients from China reported an overall variant detection rate of 78%, a value that is lower than that in European cohorts [32, 33]. These points highlight the impact of having a high pre-genetic testing probability and of utilising comprehensive test strategies and analytical approaches that are appropriate for a given population.

**Fig. 4:**
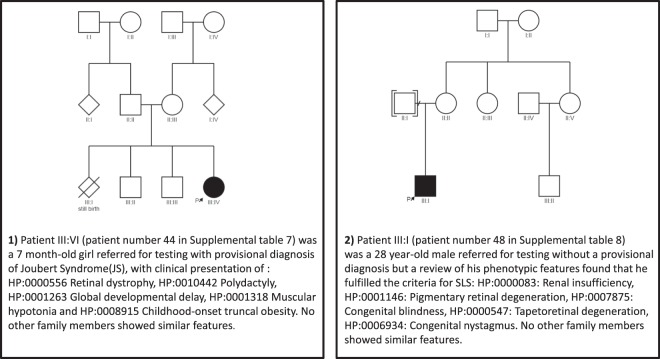
Case study describing the pedigree and clinical features of two unrelated patients with different phenotypes but were found to have the same disease-associated homozygous variant in *CEP290* gene causative of their conditions.

Our cohort of patients with ciliopathies illustrates the genetic and phenotypic overlap observed previously in this group of diseases. We had two unrelated patients with different phenotypes but who were found to have the same homozygous nonsense variant NM_025114.3:c.5668G>T p.(Gly1890*) in *CEP290* which had previously been described in Joubert syndrome patients. [25, 26, 34] (Fig. 4). It is well known that disease-associated *CEP290* variants cause a spectrum of phenotypes that vary in organ involvement and severity. Although it remains unclear why the same homozygous nonsense variant results in variable phenotypes, it has recently been hypothesised that the presence of genetic modifiers, within *CEP290* or its genetic interactors, could be one possible explanation [35].

An intriguing finding in our study was that 6 of the 51 individuals who had extra-ocular features that did not fit with known clinical conditions were found to have variants in genes that had been linked to ocular-only disease (Table 1). All these six patients had a single extra-ocular manifestation each, including four patients with DD/ID, one with ataxia, one with skeletal dysplasia and one with significant obesity in childhood. There are two plausible explanations for this finding. One is that this represents expansion of the phenotype linked to specific non-syndromic IRD genes; notably, 23 of the 78 genes in our 176 gene panel that are linked to syndromic IRD have been also described in association with isolated retinopathy. Another explanation is that two distinct genetic conditions are present in a single individual. Notably, a previous retrospective analysis of 2076 patients with various disorders who received a molecular diagnosis revealed that 4.9% of them had had diagnoses that involved more than one disease locus [36] referred to more commonly now as a ‘blended’ phenotype.

Our findings suggest that panel-based testing such as was employed here is a highly successful molecular approach for providing a genetic diagnosis in patients with IRD and recognised features of a specific multisystemic condition such as BBS and Usher syndrome. One might expect this, as if a clinical diagnosis is already suspected, referring clinician can check that the relevant gene(s) are on the testing panel. In contrast, it can be argued that genome-wide approaches (exome or genome sequencing) are more appropriate initial molecular tests in complex cases where patients have multisystemic features but lack a specific clinical diagnosis. For example, Lionel et. al, have found that WGS had a better diagnostic yield in comparison with panel tests and WES, identifying the variants found by them and variants beyond their scope and increasing the yield from 24 to 41% [37]. In addition, over time, this will save time and cost, by avoiding the need to repeat genetic tests as new genes are discovered or new symptoms emerge. As we have found in this cohort, 15% (*n* = 10) of the 65 patients who were tested on 176 gene panel would not have been diagnosed using the 105 gene panel. However, this direct comparison is unfair as the types of patients referred to each panel differed according to the genes and their associated diseases. In practice, a hybrid approach, where a whole exome or genome is sequenced but virtual panels of genes are analysed, is likely to be a cost-effective strategy that offers a compromise between maximising the yield of genetic testing and addressing complicated issues surrounding secondary findings. This has the added advantage that one can go back to the initial data and analyse a further panel of genes if clinical signs and symptoms change over time and a clinical diagnosis becomes apparent.

## Supplementary information

Supplemental_tables_SyndromicIRD_supp-tables-S1-S11

Supplemental_figures_SyndromicIRD_supp-Figure-S1-S3
