# Supplementary material for: Diagnostic yield of panel-based genetic testing in syndromic inherited retinal disease
Source: Eur J Hum Genet. 2019 Dec 13;28(5):576–86. doi: 10.1038/s41431-019-0548-5 (PMC7171123; doi:10.1038/s41431-019-0548-5)
Supplement: Supplementary file 1 — Supplemental_tables_SyndromicIRD_supp-tables-S1-S11 [file 41431_2019_548_MOESM1_ESM.docx]

Yield of panel NGS testing in syndromic IRD …

**SUPPLEMENTAL MATERIAL (TABLES)**

Supplemental table S1: diagnostic criteria for the known syndromes.

| Syndrome | Diagnostic criteria | Reference |
| --- | --- | --- |
| Usher syndrome | hearing impairment at any age with rod-cone dystrophy, with or without vestibular dysfunction | Mathur P, Yang J. Usher syndrome: hearing loss, retinal degeneration and associated abnormalities. Biochimica et Biophysica Acta (BBA)-Molecular Basis of Disease. 2015;1852(3):406-20. |
| BBS | Clinical diagnosis: Four out of six primary features or 3 primary features plus 2 secondary features.  High index of suspicion: requiring one more primary feature (i.e. has 3 primary features or 2 primary and 2 secondary)  **Primary features:** rod-cone dystrophy, polydactyly, obesity, renal anomalies, learning disabilities, and hypogonadism in males. **Secondary features:** speech disorder or delay, strabismus/cataracts/astigmatism, brachydactyly/syndactyly, developmental delay, polyuria/polydipsia (nephrogenic diabetes insipidus), ataxia/poor coordination/imbalance, mild spasticity (especially lower limbs), diabetes mellitus, dental crowding/ hypodontia/small roots/high arched palate, left ventricular hypertrophy/congenital heart disease, and hepatic fibrosis. | Beales PL, Elcioglu N, Woolf AS, Parker D, Flinter FA. New criteria for improved diagnosis of Bardet-Biedl syndrome: results of a population survey. Journal of Medical Genetics. 1999;36(6):437-46. |
| Senior-Loken syndrome | a form of abnormality of retinal pigmentation with nephronophthisis or ESRD | Ronquillo CC, Bernstein PS, Baehr W. Senior-Loken syndrome: A syndromic form of retinal dystrophy associated with nephronophthisis. Vision Research. 2012;75:88-97. |
| Cohen syndrome | Learning difficulties in addition to two out of three major features to fulfil a clinical diagnosis of Cohen syndrome: neutropenia (defined as a neutrophil count <2 x 10^-9^/mm^3^), pigmentary retinopathy, and facial dysmorphism characteristic of Cohen syndrome. | Chandler KE, Kidd A, Al-Gazali L, Kolehmainen J, Lehesjoki AE, Black GCM, et al. Diagnostic criteria, clinical characteristics, and natural history of Cohen syndrome. Journal of Medical Genetics. 2003;40(4):233-41. |
| Norrie disease | early eye involvement including vitreoretinal dysplasia and retinal detachment, with sensorineural hearing impairment, and neurological manifestations such as intellectual disability and rarely seizures | Arai E, Fujimaki T, Yanagawa A, Fujiki K, Yokoyama T, Okumura A, et al. Familial cases of Norrie disease detected by copy number analysis. Japanese Journal of Ophthalmology. 2014;58(5):448-54. |
| Joubert syndrome | developmental delay, hypotonia and characteristic Magnetic resonance imaging (MRI) findings | Bulgheroni S, D'Arrigo S, Signorini S, Briguglio M, Di Sabato ML, Casarano M, et al. Cognitive, Adaptive, and Behavioral Features in Joubert Syndrome. American Journal of Medical Genetics Part A. 2016;170(12):3115-24. |

Supplemental Table S2: Genes and transcripts tested with the 105 inherited retinal disease gene panel

| **HGNC** | **Transcript** | **HGNC** | **Transcript** | **HGNC** | **Transcript** | **HGNC** | **Transcript** |
| --- | --- | --- | --- | --- | --- | --- | --- |
| *ABCA4* | NM_000350.2 | *CRB1* | NM_201253.2 | *NR2E3* | NM_014249.2 | *RIMS1* | NM_001168410.1 |
| *ADAM9* | NM_003816.2 | *CRX* | NM_000554.4 | *NRL* | NM_006177.3 | *RLBP1* | NM_000326.4 |
| *AIPL1* | NM_014336.3 | *DFNB31* | NM_015404.3 | *OTX2* | NM_021728.2 | *ROM1* | NM_000327.3 |
| *ARL6* | NM_032146.3 | *DHDDS* | NM_024887.2 | *PCDH15* | NM_001142763.1 | *RP1* | NM_006269.1 |
| *BBS1* | NM_024649.4 | *EFEMP1* | NM_001039348.2 | *PCDH15* | NM_001142769.1 | *RP1L1*** | NM_178857.5 |
| *BBS10* | NM_024685.3 | *ELOVL4* | NM_022726.3 | *PCDH15* | NM_001142770.1 | *RP2* | NM_006915.2 |
| *BBS12* | NM_001178007.1 | *EYS* | NM_001142800.1 | *PCDH15* | NM_001142771.1 | *RP9* | NM_203288.1 |
| *BBS2* | NM_031885.3 | *FAM161A* | NM_001201543.1 | *PDE6A* | NM_000440.2 | *RPE65* | NM_000329.2 |
| *BBS4* | NM_033028.3 | *FSCN2* | NM_001077182.2 | *PDE6B* | NM_000283.3 | *RPGR** | NM_001034853.1 |
| *BBS5* | NM_152384.2 | *FZD4* | NM_012193.3 | *PDE6C* | NM_006204.3 | *RPGRIP1* | NM_020366.3 |
| *BBS7* | NM_176824.2 | *GNAT2* | NM_005272.3 | *PDE6G* | NM_002602.3 | *RS1* | NM_000330.3 |
| *BBS7* | NM_018190.3 | *GPR98* | NM_032119.3 | *PITPNM3* | NM_031220.3 | *SAG* | NM_000541.4 |
| *BBS9* | NM_198428.2 | *GUCA1A* | NM_000409.3 | *PRCD* | NM_001077620.2 | *SEMA4A* | NM_022367.3 |
| *BEST1* | NM_004183.3 | *GUCA1B* | NM_002098.5 | *PROM1* | NM_006017.2 | *SNRNP200* | NM_014014.4 |
| *C1QTNF5* | NM_015645.3 | *GUCY2D* | NM_000180.3 | *PRPF3* | NM_004698.2 | *SPATA7* | NM_018418.4 |
| *C2orf71* | NM_001029883.1 | *IDH3B* | NM_006899.2 | *PRPF31* | NM_015629.3 | *TEAD1* | NM_021961.5 |
| *CA4* | NM_000717.3 | *IDH3B* | NM_174855.1 | *PRPF6* | NM_012469.3 | *TIMP3* | NM_000362.4 |
| *CACNA2D4* | NM_172364.4 | *IMPDH1* | NM_000883.3 | *PRPF8* | NM_006445.3 | *TOPORS* | NM_005802.4 |
| *CDH23* | NM_022124.5 | *IMPG2* | NM_016247.3 | *PRPH2* | NM_000322.4 | *TRIM32* | NM_012210.3 |
| *CDHR1* | NM_001171971.1 | *KCNV2* | NM_133497.3 | *RAX2* | NM_032753.3 | *TTC8* | NM_144596.2 |
| *CDHR1* | NM_033100.2 | *KLHL7* | NM_001031710.2 | *RBP3* | NM_002900.2 | *TULP1* | NM_003322.3 |
| *CEP290**** | NM_025114.3 | *LCA5* | NM_181714.3 | *RD3* | NM_183059.2 | *UNC119* | NM_005148.3 |
| *CERKL* | NM_001030311.2 | *LRAT* | NM_004744.3 | *RDH12* | NM_152443.2 | UNC119 | NM_054035.2 |
| *CHM* | NM_000390.2 | *LRP5* | NM_002335.2 | *RDH5* | NM_001199771.1 | *USH1C* | NM_005709.3 |
| *CLRN1* | NM_052995.2 | *MERTK* | NM_006343.2 | *RGR* | NM_002921.3 | *USH1C* | NM_153676.3 |
| *CLRN1* | NM_001195794.1 | *MKKS* | NM_018848.2 | *RGS9* | NM_003835.3 | *USH1G* | NM_173477.2 |
| *CNGA1* | NM_001142564.1 | *MKS1* | NM_017777.3 | *RGS9* | NM_001165933.1 | *USH2A* | NM_206933.2 |
| *CNGA3* | NM_001298.2 | *MKS1* | NM_001165927.1 | *RHO* | NM_000539.3 | *ZNF513* | NM_144631.5 |
| *CNGB1* | NM_001297.4 | *MYO7A* | NM_000260.3 | *RIMS1* | NM_014989.4 |  |  |
| *CNGB3* | NM_019098.4 | *NDP* | NM_000266.3 | *RIMS1* | NM_001168407.1 |  |  |

**Notes:**

(1) For some genes the analysis of multiple transcripts is required.

(2)* For *RPGR* it is not possible to obtain high quality read for exon ORF15.

(3)** Analysis of the coding region between c.4000- c.7000 of the RP1L1 gene is not included

(4)***Testing of the common intron 26 mutation c.2991+1655A>G in CEP290 is included in this analysis

Supplemental Table S3: Genes and transcripts tested with the 176 inherited retinal disease gene panel (July 2014)

| ***HGNC*** | **Transcript** | ***HGNC*** | **Transcript** | ***HGNC*** | **Transcript** | ***HGNC*** | **Transcript** | ***HGNC*** | **Transcript** | ***HGNC*** | **Transcript** |
| --- | --- | --- | --- | --- | --- | --- | --- | --- | --- | --- | --- |
| *ABCA4** | NM_000350.2 | *CDHR1* | NM_033100.3 | *GPR179* | NM_001004334.3 | *MVK* | NM_000431.2 | *PRPF4* | NM_004697.4 | *SNRNP200* | NM_014014.4 |
| *ABHD12* | NM_001042472.2 | *CEP164* | NM_014956.4 | *GPR98* | NM_032119.3 | *MYO7A* | NM_000260.3 | *PRPF6* | NM_012469.3 | *SPATA7* | NM_018418.4 |
| *ACBD5* | NM_145698.3 | *CEP290** | NM_025114.3 | *GRM6* | NM_000843.3 | *NDP* | NM_000266.3 | *PRPF8* | NM_006445.3 | *TEAD1* | NM_021961.5 |
| *ADAM9* | NM_003816.2 | *CERKL* | NM_001030311.2 | *GUCA1A* | NM_000409.3 | *NEK2* | NM_002497.3 | *PRPH2* | NM_000322.4 | *TIMP3* | NM_000362.4 |
| *ADAMTS18* | NM_199355.2 | *CHM* | NM_000390.2 | *GUCA1B* | NM_002098.5 | *NMNAT1* | NM_022787.3 | *RAB28* | NM_001017979.2 | *TMEM237* | NM_001044385.2 |
| *AHI1* | NM_001134832.1 | *CIB2* | NM_006383.3 | *GUCY2D* | NM_000180.3 | *NPHP1* | NM_000272.3 | *RAX2* | NM_032753.3 | *TOPORS* | NM_005802.4 |
| *AHI1* | NM_017651.4 | *CLN3* | NM_000086.2 | *HARS* | NM_002109.5 | *NPHP3* | NM_153240.4 | *RBP3* | NM_002900.2 | *TRIM32* | NM_012210.3 |
| *AIPL1* | NM_014336.4 | *CLRN1* | NM_052995.2 | *HMX1* | NM_018942.2 | *NPHP4* | NM_015102.4 | *RBP4* | NM_006744.3 | *TRPM1* | NM_002420.4 |
| *ARL2BP* | NM_012106.3 | *CLRN1* | NM_001195794.1 | *IDH3B* | NM_006899.3 | *NR2E3* | NM_014249.3 | *RD3* | NM_183059.2 | *TSPAN12* | NM_012338.3 |
| *ARL6* | NM_032146.4 | *CNGA1* | NM_001142564.1 | *IDH3B* | NM_174855.2 | *NRL* | NM_006177.3 | *RDH12* | NM_152443.2 | *TTC8* | NM_144596.3 |
| *BBIP1* | NM_001195306.1 | *CNGA3* | NM_001298.2 | *IFT140* | NM_014714.3 | *NYX* | NM_022567.2 | *RDH5* | NM_001199771.1 | *TUB* | NM_177972.2 |
| *BBS1* | NM_024649.4 | *CNGB1* | NM_001297.4 | *IMPDH1* | NM_000883.3 | *OAT* | NM_000274.3 | *RGR* | NM_002921.3 | *TULP1* | NM_003322.4 |
| *BBS10* | NM_024685.3 | *CNGB3* | NM_019098.4 | *IMPG1* | NM_001563.3 | *OFD1** | NM_003611.2 | *RGS9* | NM_003835.3 | *UNC119* | NM_005148.3 |
| *BBS12* | NM_001178007.1 | *CNNM4* | NM_020184.3 | *IMPG2* | NM_016247.3 | *OTX2* | NM_021728.3 | *RGS9* | NM_001165933.1 | *UNC119* | NM_054035.2 |
| *BBS2* | NM_031885.3 | *CRB1* | NM_201253.2 | *INPP5E* | NM_019892.4 | *PANK2* | NM_153638.2 | *RHO* | NM_000539.3 | *USH1C* | NM_005709.3 |
| *BBS4* | NM_033028.4 | *CRX* | NM_000554.4 | *INVS* | NM_014425.3 | *PCDH15* | NM_001142763.1 | *RIMS1* | NM_014989.5 | *USH1C* | NM_153676.3 |
| *BBS5* | NM_152384.2 | *CSPP1* | NM_024790.6 | *IQCB1* | NM_001023570.2 | *PCDH15* | NM_001142769.1 | *RIMS1* | NM_001168407.1 | *USH1G* | NM_173477.4 |
| *BBS7* | NM_176824.2 | *CYP4V2* | NM_207352.3 | *ITM2B* | NM_021999.4 | *PCDH15* | NM_001142770.1 | *RIMS1* | NM_001168410.1 | *USH2A** | NM_206933.2 |
| *BBS9* | NM_198428.2 | *DFNB31* | NM_015404.3 | *KCNJ13* | NM_002242.4 | *PCDH15* | NM_001142771.1 | *RLBP1* | NM_000326.4 | *VCAN* | NM_004385.4 |
| *BEST1* | NM_004183.3 | *DHDDS* | NM_024887.3 | *KCNV2* | NM_133497.3 | *PCYT1A* | NM_005017.2 | *ROM1* | NM_000327.3 | *VPS13B* | NM_017890.4 |
| *C1QTNF5* | NM_015645.4 | *DTHD1* | NM_001136536.4 | *KIAA1549* | NM_001164665.1 | *PDE6A* | NM_000440.2 | *RP1* | NM_006269.1 | *WDR19* | NM_025132.3 |
| *C2orf71* | NM_001029883.2 | *DTHD1* | NM_001170700.2 | *KIAA1549* | NM_020910.2 | *PDE6B* | NM_000283.3 | *RP1L1****^**^*** | NM_178857.5 | *ZNF423* | NM_015069.3 |
| *C2ORF86/(WDPCP)* | NM_015910.4 | *EFEMP1* | NM_001039348.2 | *KIF11* | NM_004523.3 | *PDE6C* | NM_006204.3 | *RP2* | NM_006915.2 | *ZNF513* | NM_144631.5 |
| *C8ORF37* | NM_177965.3 | *ELOVL4* | NM_022726.3 | *KLHL7* | NM_001031710.2 | *PDE6G* | NM_002602.3 | *RP9* | NM_203288.1 |  |  |
| *C21orf2* | NM_004928.2 | *EMC1* | NM_015047.2 | *LCA5* | NM_181714.3 | *PEX1* | NM_000466.2 | *RPE65* | NM_000329.2 |  |  |
| *CA4* | NM_000717.3 | *EYS* | NM_001142800.1 | *LRAT* | NM_004744.4 | *PEX2/(PXMP3)* | NM_000318.2 | *RPGR****^***^*** | NM_001034853.1 |  |  |
| *CABP4* | NM_145200.3 | *FAM161A* | NM_001201543.1 |  |  | *PEX7* | NM_000288.3 | *RPGRIP1* | NM_020366.3 |  |  |
| *CACNA1F* | NM_005183.3 | *FLVCR1* | NM_014053.3 | *LRP5* | NM_002335.3 | *PHYH* | NM_006214.3 | *RPGRIP1L* | NM_015272.2 |  |  |
| *CACNA2D4* | NM_172364.4 | *FSCN2* | NM_001077182.2 | *LZTFL1* | NM_020347.3 | *PITPNM3* | NM_031220.3 | *RS1* | NM_000330.3 |  |  |
| *CAPN5* | NM_004055.4 | *FZD4* | NM_012193.3 | *MERTK* | NM_006343.2 | *PLA2G5* | NM_000929.2 | *SAG* | NM_000541.4 |  |  |
| *CC2D2A* | NM_001080522.2 | *GNAT1* | NM_000172.3 | *MFRP* | NM_031433.3 | *PRCD* | NM_001077620.2 | *SDCCAG8* | NM_006642.3 |  |  |
| *CDH3* | NM_001793.4 | *GNAT2* | NM_005272.3 | *MKKS* | NM_018848.3 | *PROM1* | NM_006017.2 | *SEMA4A* | NM_022367.3 |  |  |
| *CDH23* | NM_022124.5 | *GNPTG* | NM_032520.4 | *MKS1* | NM_017777.3 | *PRPF3* | NM_004698.2 | *SLC24A1* | NM_004727.2 |  |  |
| *CDHR1* | NM_001171971.2 | *GPR125* | NM_145290.3 | *MKS1* | NM_001165927.1 | *PRPF31* | NM_015629.3 | *SLC24A1* | NM_001254740.1 |  |  |

**Notes:**

(1) The latest versions of the transcripts are used during analysis.

(2)For some genes the analysis of multiple transcripts is required.

(3)*Testing for the following intronic mutations are included in this analysis:

- *CEP290* c.2991+1655A>G

- *USH2A* c.7595-2144A>G

- *OFD1* c.935+706A>G

- *ABCA4* c.5196+1056A>G, c.5196+1137G>A, c.5196+1216C>A, c.4539+2001G>A, c.4539+2028C>T & ABCA4 c.5461-10T>C

(4)^**^Analysis of the coding region of exon 4 of the *RP1L1* gene is not included

(5)^***^ For *RPGR* it is not possible to obtain high quality read for exon ORF15.

| HP number and term | Pts | HP number and term | pts | HP number and term | pts |
| --- | --- | --- | --- | --- | --- |
| HP:0000510 Rod cone dystrophy | 54 | HP:0008527 Congenital sensorineural hearing impairment | 15 | HP:0001249 Intellectual disability; HP:0010864 Intellectual disability, severe; | 20 |
| HP:0000556 Retinal dystrophy or HP:0000488 Retinopathy; HP:0007703 Abnormality of retinal pigmentation; HP:0007814 Retinal pigment epithelial mottling; | 29 | HP:0000365 Hearing impairment/loss; HP:0001730 Progressive hearing impairment; HP:0012715 Profound hearing impairment; | 12 | HP:0001263 Global developmental delay; HP:0012736 Profound global developmental delay; | 18 |
| HP:0000548 Cone/cone-rod dystrophy | 10 | HP:0008615 Adult onset sensorineural hearing impairment | 8 | HP:0002194 Delayed gross motor development; HP:0001270 Motor delay; HP:0002421: poor head control; | 7 |
| HP:0000545 Myopia; HP:0011003 Severe Myopia | 8 | HP:0000399 Prelingual sensorineural hearing impairment | 4 | HP:0000750 Delayed speech and language development | 6 |
| HP:0000662 Nyctalopia | 6 | HP:0000407: Sensorineural hearing impairment; | 3 | HP:0001318 Muscular hypotonia  HP:0007754 Macular dystrophy; HP:0008947: Infantile muscular hypotonia; HP:0001319 Neonatal hypotonia; HP:0008936 Muscular hypotonia of the trunk; | 5 |
| HP:0007875: Congenital blindness; HP:0000505: Visual Impairment; HP:0007663 Reduced visual acuity; | 5 | HP:0008596 Postlingual sensorineural hearing impairment | 2 | HP:0001513 Obesity; HP:0001956 Truncal obesity; | 8 |
| HP:0000518 Cataract; HP:0000519: Congenital cataract  HP:0007787 Posterior subcapsular cataract; | 5 | HP:0011474 childhood onset sensorineural Hearing impairment | 2 | HP:0025502 Overweight; HP:0004324 Increased body weight; | 5 |
| HP:0006934: Congenital nystagmus; HP:0000639 Nystagmus; HP:0000666 Horizontal nystagmus | 5 | HP:0001751 Vestibular dysfunction; | 1 | HP:0001510 Growth delay; HP:0004322 Short stature; | 2 |
| HP:0007973:Retinal dysplasia; HP:0000541:Retinal detachment; HP:0007773 Vitreoretinopathy  (Bilateral vitreo-retinal dysplasia) | 4 |  |  | HP:0001508 Failure to thrive; HP:0008897: Postnatal growth retardation; | 2 |
| HP:0007754 Macular dystrophy; HP:0007401 Macular atrophy | 3 | HP:0001999 Abnormal facial shape; HP:0000280: Coarse facial features; | 9 | HP:0000098: Tall stature | 1 |
| HP:0000543 Optic disc pallor HP:0000609 optic nerve hypoplasia | 2 | HP:0000252 Microcephaly; | 9 |  |  |
| HP:0000547: Tapetoretinal degeneration; | 1 | HP:0010442 Polydactyly; HP:0100259 Postaxial polydactyly | 9 | HP:0000819 Diabetes mellitus | 2 |
| HP:0007642 Congenital stationary night blindness; | 1 | HP:0001156 Brachydactyly; | 3 | HP:0000821 Hypothyroidism; | 1 |
| HP:0007830: Adult-onset night blindness; | 1 | HP:0001159 Syndactyly | 2 |  |  |
| HP:0007957 Corneal opacity; | 1 | HP:0002652 Skeletal dysplasia; HP:0002942:Thoracic kyphosis; | 2 | HP:0000083 Renal insufficiency;  HP:0003774 End stage renal failure; | 5 |
| HP:0000532 Chorioretinal abnormality | 1 | HP:0005681 Juvenile rheumatoid arthritis; HP:0002758 Osteoarthritis; | 2 | HP:0000090 Nephronophthisis; | 1 |
| HP:0008499 high-grade hypermetropia, | 1 | HP:0001166: Arachnodactyly; | 1 | HP:0000105 Enlarged kidney | 1 |
| HP:0000483 Astigmatism | 1 | HP:0001382 Joint hypermobility; | 1 | HP:0000010:Recurrent urinary tract infections; | 1 |
|  |  | HP:0010176 Curved phalanges of the toes | 1 |  |  |
| HP:0000718:Aggressive behaviour; HP:0000708 Behavioural abnormality; HP:0100716 Self-injurious behaviour; | 4 | HP:0000939 Osteoporosis; | 1 | HP:0002019:Constipation; | 2 |
| HP:0000729 Autism spectrum disorder; HP:0000717 Autism; | 3 |  |  | defect;HP:0000023 Inguinal hernia; | 1 |
|  |  | HP:0002783 Recurrent lower respiratory tract infections; | 3 | HP:0000164 Abnormality of the dentition; | 1 |
| HP:0001250: Seizures | 6 | HP:0001875 Neutropenia; | 1 | HP:0009128 Aplasia/Hypoplasia involving the musculature of the extremities; | 1 |
| HP:0001251 Cerebellar ataxia; HP:0001251 Ataxia; | 4 | HP:0002099 Asthma | 1 | HP:0000054 Micropenis | 1 |
| HP:0002283 Global brain atrophy; HP:0001272 Cerebellar atrophy; | 2 |  |  | HP:0430015 Abnormality of pharyngeal musculature; | 1 |
| HP:0001305 Dandy-Walker malformation; | 1 | HP:0001650 Aortic valve stenosis; HP:0001629 Ventricular septal defect | 2 | HP:0011968 Feeding difficulties; | 1 |
| HP:0007370 Aplasia/Hypoplasia of the corpus callosum; | 1 | HP:0001433: Hepatosplenomegaly; | 1 | HP:0004464 Postauricular pits; | 1 |
| HP:0002493 Upper motor neuron dysfunction; | 1 | HP:0001410 Decreased liver function; | 1 | HP:0100540 Palpebral edema; | 1 |
| HP:0001789 Hydrops fetalis; | 1 | HP:0000822 Hypertension; HP:0001635 Congestive heart failure | 1 | HP:0010978 Abnormality of immune system physiology; | 1 |
| HP:0002401 Stroke-like episode | 1 | HP:0001004 Lymphoedema; | 1 | HP:0500001: Body odour; | 1 |
|  |  | HP:0030984 Abnormal serum bile acid concentration; | 1 | HP:0100502 Vitamin B12 deficiency | 1 |

Supplemental Table S4: Human Phenotype Ontology (HPO) terms of phenotypes of all 106 patients and number of patients that had the phenotype.

**Supplemental Table S5: Details of the 33 Usher patients included in this study and corresponding genetic variants identified as probably or possibly causal of their clinical presentation by IRD panel testing.**

| Pt. no. | Number of genes tested | Gender | Age at referral for testing | Phenotype (HPO terms) | Variants probably or possibly accounting  for clinical presentation | Genetic diagnosis | Segregation analysis |  |
| --- | --- | --- | --- | --- | --- | --- | --- | --- |
| 1 | 105 | F | 0.4 | HP:0008527 Congenital sensorineural hearing impairment; HP:0002194 Delayed gross motor development;  HP:0000510 Rod-cone dystrophy | MYO7A  NM_000260.3:c.[6025del];[4115T>G]  p.[(Ala2009Profs*32)];[(Val1372Gly)] | Probable | Trans  (Mot, Fat) | c.6025del: Bharadwaj (2000) Exp Eye Res 71: 173 PubMed: 10930322  c.4115T>G :same patient: Ellingford (2016) Ophthalmology 123: 1143 PubMed: 26872967 |
| 2 | 105 | F | 5.5 | HP:0008527 Congenital sensorineural hearing impairment; HP:0000510 Rod-cone dystrophy | MYO7A  NM_000260.3:c.[5899C>T];[1135G>A] p.[(Arg1967*)];[Gly379Arg)] | Probable | Trans  (Mot, Fat) | c.5899C>T: Bonnet (2016) Eur J Hum Genet 24: 1730 PubMed: 27460420  c.1135G>A: same patient: Ellingford (2016) J Med Genet 53: 761 PubMed: 27208204 |
| 3 | 176 | F | 5.8 | HP:0008527 Congenital sensorineural hearing impairment;  HP:0000545 Myopia;  HP:0000662 Nyctalopia;  HP:0000510 Rod-cone dystrophy | USH2A  NM 206933.2: c.[5776+1G>A];[5776+1G>A] | Probable |  | Patel (2016) Genet Med 18: 554 PubMed: 26355662 |
| 4 | 105 | F | 8.2 | HP:0000399 Prelingual sensorineural hearing impairment; HP:0000510 Rod-cone dystrophy | PCDH15  NM_001142771.1:  c.[2986C>T];[-189197_610-5166del] (Exons 2-7 deletion)  p.[(Arg996*)];[?] | Probable | Trans (Mot) | Same patient reported by our group: Ellingford (2016) Ophthalmology 123: 1143 PubMed: 26872967 |
| 5 | 176 | F | 11.3 | HP:0008527 Congenital sensorineural hearing impairment; HP:0000510 Rod-cone dystrophy | USH1G  NM_173477.2:c.[511G>T];[511G>T] p.[(Glu171*)];[(Glu171*)] | Probable |  | Same patient: Published by our group: Taylor (2017) Ophthalmology 124: 985 PubMed: 28341476 |
| 6 | 176 | M | 14.4 | HP:0008527 Congenital sensorineural hearing impairment; HP:0000488 Retinopathy | MYO7A  NM_000260.3: c.[4996_4997del];[4996_4997del] p.[(Ser1666Cysfs*54)];[(Ser1666Cysfs*54)] | Probable |  | Same patient: Published by our group: Taylor (2017) Ophthalmology 124: 985 PubMed: 28341476 |
| 7 | 176 | F | 15.3 | HP:0008527 Congenital sensorineural hearing impairment; HP:0000510 Rod-cone dystrophy | USH2A NM_206933.2:c.[5776+1G>A(;)(11047+1_11048-1)_(11711+1_11712-1)dup]  exons 57-60 (3 copies) | Probable |  | 5776+1G>A: Patel (2016) Genet Med 18: 554 PubMed: 26355662  Large dup.: Lenassi (2015) Eur J Hum Genet 23: 1318 PubMed: 25649381 |
| 8 | 176 | M | 16.5 | HP:0008527: Congenital sensorineural hearing impairment; HP:0000510: Rod-cone dystrophy;  HP:0001249: Intellectual disability | PCDH15  NM_001142771.1: c.[1942C>T];[1942C>T]  p.[(Arg648*)]; [(Arg648*)] | Probable |  | Novel |
| 9 | 176 | F | 24.9 | HP:0012714 Severe hearing impairment;  HP:0000399 Prelingual sensorineural hearing impairment; HP:0000510: Rod-cone dystrophy; | USH2A  NM_206933.2:c.[6862G>T];[3407G>A]  p.[(Glu2288*)];[(Ser1136Asn)] | Probable |  | c.6862G>T: Yan (2009) J Hum Genet 54: 732 PubMed: 19881469  c.3407G>A: Le Quesne Stabej (2012) J Med Genet 49: 27 PubMed: 22135276 |
| 10 | 105 | F | 27.0 | HP:0008527 Congenital sensorineural hearing impairment; HP:0000662 Nyctalopia;  HP:0001133 Constriction of peripheral visual field; HP:0000510: Rod-cone dystrophy; | USH2A  NM_206933.2:c.[4645C>T(;)3158-2A>G]  p.(Arg1549*)];[?] | Probable |  | same patient: Ellingford (2016) J Med Genet 53: 761 PubMed: 27208204  c.4645C>T: Baux (2007) Hum Mutat 28: 781 PubMed: 17405132 |
| 11 | 176 | F | 30.6 | HP:0008527 Congenital sensorineural hearing impairment; HP:0000510 Rod cone dystrophy | USH2A  NM_206933.2:c.[6657+1G>A(;)6657+1G>A] | Probable |  | c.6657+1G>A : Bonnet (2016) Eur J Hum Genet 24: 1730 PubMed: 27460420 |
| 12 | 176 | F | 30.7 | HP:0008615 Adult onset sensorineural hearing impairment; HP:0000510 Rod cone dystrophy | CLRN1 (USH3A)  NM_001195794.1:c.[118T>G];[65T>A]  p.[(Cys40Gly)];[(Leu22His)] | Probable |  | c. 118T>G: Aller (2004) Clin Genet 66: 525 PubMed: 15521980  c.65T>A: Novel |
| 13 | 176 | M | 33.9 | HP:0000399 Prelingual sensorineural hearing impairment; HP:0000510 Rod cone dystrophy | USH2A  NM_206933.2:c.[1859G>T];[14803C>T]  p.[(Cys620Phe)];[(Arg4935*)] | Probable |  | c.1859G>T :Cremers (2007) J Med Genet 44: 153 PubMed: 16963483  c.14803C>T: Baux (2007) Hum Mutat 28: 781 PubMed: 17405132 |
| 14 | 105 | M | 48.2 | HP:0008615 Adult onset sensorineural hearing impairment; HP:0000510 Rod cone dystrophy | USH2A  NM_206933.2:c.[6446C>A];[6326-3582_6658-1028del] (Exons 33,34 deletion) p.(Pro2149Gln)];[?] | Probable |  | Same patient reported by our group: Ellingford (2016) Ophthalmology 123: 1143 PubMed: 26872967  c.6446C>A: Carss (2017) Am J Hum Genet 100: 75 PubMed: 28041643 |
| 15 | 176 | F | 49.6 | HP:0008527 Congenital sensorineural hearing impairment; HP:0000556 Retinal dystrophy;  HP:0007787 Posterior subcapsular cataract | USH2A  NM_206933.2:c.[2299del];[6446C>A]  p.[(Glu767Serfs*21)];[(Pro2149Gln)] | Probable |  | c. 2299del:Eudy (1998) Science 280: 1753 PubMed: 9624053.  c.6446C>A: Carss (2017) Am J Hum Genet 100: 75 PubMed: 28041643 |
| 16 | 176 | F | 52.1 | HP:0008527 Congenital sensorineural hearing impairment;  HP:0007675 Progressive night blindness;  HP:0007994 Peripheral visual field loss H  P:0000510 Rod cone dystrophy | USH2A  NM_206933.2: c.[12067-1G>A];[2209C>T]  p.[?];[(Arg737*)] | Probable |  | c. 12067-1G>A: Auslender (2008) Genet Test 12: 289 PubMed: 18452394  c.2209C>T Huang (2015) Genet Med 17: 271 PubMed: 25356976 |
| 17 | 105 | F | 21.6 | HP:0012712 Mild hearing impairment;  HP:0000510 Rod cone dystrophy | CLRN1 (USH3A)  NM_001195794.1:c.[144T>G];[144T>G] p.[(Asn48Lys)];[(Asn48Lys)] | Probable |  | c. 144T>G: Adato (2002) Eur J Hum Genet 10: 339 PubMed: 12080385 |
| 18 | 105 | F | 25.2 | HP:0000365 Hearing impairment/loss;  HP:0000556 Retinal dystrophy | USH2A  NM_206933.2:c.[2299del];[4321G>T] p.[(Glu767Serfs*21)];[(Glu1441*)] | Probable | Trans  (Mot, Fat) | c.2299del:Eudy (1998) Science 280: 1753 PubMed: 9624053.  c.4321G>T: O'Sullivan (2012) J Med Genet 49: 322 PubMed: 22581970 |
| 19 | 105 | M | 27.3 | HP:0000365 Hearing impairment/loss;  HP:0000556 Retinal dystrophy | USH2A  NM_206933.2:c.[1558del];[1558del]  p.[(Cys520Alafs*71)]; [(Cys520Alafs*71)] | Probable |  | Same patient: Ellingford (2016) J Med Genet 53: 761 PubMed: 27208204 |
| 20 | 176 | F | 29.1 | HP:0008527 Congenital sensorineural hearing impairment;  HP:0000510 Rod cone dystrophy | ADGRV1(GPR98)  NM_032119.3:c.[9748+2T>C];  [6962_6963del]  p.[?];[(Val2321Alafs*4)] | Probable |  | c.9748+2T>C: Novel  c.6962_6963del: Le Quesne Stabej (2012) J Med Genet 49: 27 PubMed: 22135276 |
| 21 | 176 | F | 34.7 | HP:0000365 Hearing impairment/loss;  HP:0000510 Rod cone dystrophy | USH2A  NM_206933.2:c.[11699A>G];[9371+1G>C]  p.[(Tyr3900Cys)];[?] | Probable |  | c. 11699A>G: Novel  c.9371+1G>C: Le Quesne Stabej (2012) J Med Genet 49: 27 PubMed: 22135276 |
| 22 | 105 | M | 44.4 | HP:0000365 Hearing impairment/loss; HP:0000662 Nyctalopia;  HP:0007663 Reduced visual acuity;  HP:0000510 Rod cone dystrophy | USH2A  NM_206933.2:c.[1859G>T];[2139C>T]  p.[(Cys620Phe)];[(Gly713Gly)] | Probable |  | 1859G>T, c. 2139C>T: same patient: Ellingford (2016) J Med Genet 53: 761 PubMed: 27208204 |
| 23 | 176 | F | 24.4 | HP:0008527: Congenital sensorineural hearing impairment; HP:0000510 Rod cone dystrophy | ADGRV1(GPR98)  NM_032119.3:c.[13232-1G>A];[ 13232-1=]  No relevant CNV detected | Possible |  | c.13232-1G>A: Novel |
| 24 | 176 | F | 32.3 | HP:0008527: Congenital sensorineural hearing impairment; HP:0000510 Rod cone dystrophy | ADGRV1(GPR98)  NM_032119.3:c.[2680del];[2680=]  No CNV detected | Possible |  | c.2680del: Novel |
| 25 | 105 | F | 40.1 | HP:0008610: Infantile sensorineural hearing impairment; HP:0000510 Rod cone dystrophy | ADGRV1(GPR98)  NM_032119.3:c.[16079-1455_16196+155del];[=] (exon 75 deletion)  WGS:  NM_032119.3:c.[1239-8C>G];[16079-1455_16196+155del] | Possible | Trans (Mot) | same patient: Ellingford (2016) Ophthalmology 123: 1143 PubMed: 26872967 |
| 26 | 105 | F | 40.7 | HP:0008527: Congenital sensorineural hearing impairment; HP:0007830 Adult-onset night blindness;  HP:0000510 Rod cone dystrophy | Panel:  USH2A  NM_206933.2:c.[4474G>T];[?] p.[(Glu1492*)];[?]  WGS detected USH2A NM_206933.2:c.[5614=];[5614delinsTTAACTTGGCAT] | Possible | Trans (Mot) | c.4474G>T: Bernal (2005) Clin Genet 68: 204 PubMed: 16098008  c.5614delinsTTAACTTGGCAT: same patient: Ellingford (2016) Ophthalmology 123: 1143 PubMed: 26872967 |
| 27 | 176 | F | 44.6 | HP:0008615 Adult onset sensorineural hearing impairment; HP:0000510 Rod cone dystrophy;  HP:0000662 Nyctalopia;  HP:0001123 Visual field defect | MYO7A  NM_000260.3:c.[1623dup];[1623=]  p.[(Lys542Glnfs*5)];[=]  No relevant CNV detected | Possible |  | c.1623dup: Bharadwaj (2000) Exp Eye Res 71: 173 PubMed: 10930322 |
| 28 | 105 | F | 42.0 | HP:0008615 Adult onset sensorineural hearing impairment; HP:0000510: Rod-cone dystrophy; HP:0002076: Migraine; HP:0001250: Seizures | No Pathogenic Mutation Identified.  No CNV | Unknown |  |  |
| 29 | 105 | M | 29.3 | HP:0008596 Postlingual sensorineural hearing impairment; HP:0000510: Rod-cone dystrophy | No pathogenic mutation detected.  No CNV | Unknown |  |  |
| 30 | 105 | F | 58.5 | HP:0000407: Sensorineural hearing impairment;  HP:0000556 Retinal dystrophy;  HP:0002283 Global brain atrophy | No pathogenic mutation detected.  No CNV | Unknown |  |  |
| 31 | 105 | M | 58.5 | HP:0000510 Rod-cone dystrophy;  HP:0000365 Hearing impairment/loss; | No pathogenic mutation identified by NGS panel.  WGS identified homozygous deletion of exons 1 to 7 of MERTK  NM_006343.2:c.[(?_-1)_(1144+1_1145-1)del];[(?_-1)_(1144+1_1145-1)del] | Autosomal recessive Rod-cone dystrophy |  | Novel |
| 32 | 176 | F | 15.0 | HP:0000365 Hearing impairment/loss;  HP:0000510 Rod-cone dystrophy | No pathogenic mutation identified.  No CNV detected | Unknown |  |  |
| 33 | 176 | F | 40.3 | HP:0012715 Profound hearing impairment;  HP:0000510 Rod cone dystrophy | No pathogenic mutation detected.  No CNV detected | Unknown |  |  |

M=male, F=female, Mot=mother tested, Fat=Father tested

Supplemental Table S6: Details of the 10 Bardet-Biedl syndrome (BBS) patients included in this study and corresponding genetic variants identified as probably or possibly causal of their clinical presentation by IRD panel testing.

| Pt. no. | Number of genes tested | Gender | Age at referral for testing | Phenotype (HPO terms) | Fulfils diagnostic criteria for BBS ? | Variants probably or possibly accounting for clinical presentation | Genetic diagnosis | Segregation Analysis |  |
| --- | --- | --- | --- | --- | --- | --- | --- | --- | --- |
| 34 | 105 | F | 36.8 | HP:0000510: Rod-cone dystrophy; HP:0010442 Polydactyly; HP:0008915 Childhood-onset truncal obesity; HP:0012434 Delayed social development; HP:0010176 Curved phalanges of the toes; | No, highly suspected (3 primary and 1 secondary) | BBS1  NM_024649.4:  c.[1169T>G];[1169T>G]  p.[(Met390Arg)];[(Met390Arg)] | probable | Homozygous (Mot, Fat) | Mykytyn (2002) Nat Genet 31: 435 PubMed: 12118255  Ashkinadze (2013) Clin Genet 83: 553 PubMed: 22998390 |
| 35 | 176 | M | 41.3 | HP:0000510 Rod-cone dystrophy; HP:0010442 Polydactyly; HP:0001249 Intellectual disability; | No, highly suspected (3 primary) | BBS1  NM_024649.4:  c.[1169T>G];[1169T>G] p.[(Met390Arg)];[(Met390Arg)] | probable |  | Mykytyn (2002) Nat Genet 31: 435 PubMed: 12118255  Ashkinadze (2013) Clin Genet 83: 553 PubMed: 22998390 |
| 36 | 176 | F | 22.1 | HP:0000510: Rod-cone dystrophy; HP:0001256 Intellectual disability, mild; HP:0011098: Verbal dyspraxia; HP:0001270: Motor delay; HP:0001156 Brachydactyly; HP:0001159 Syndactyly; HP:0000483 Astigmatism; HP:0011003 Severe Myopia; HP:0000098: Tall stature; HP:0025502 Overweight; | No, highly suspected (2 primary and 2 secondary) | BBS1  NM_024649.4:  c.[1169T>G];[1514_1515del]  p.[(Met390Arg)];[(Leu505Profs*52)] | Probable | Trans  (Mot, Fat) | Mykytyn (2002) Nat Genet 31: 435 PubMed: 12118255  c. 1514_1515del: Mykytyn (2003) Am J Hum Genet 72, 429 |
| 37 | 176 | M | 11.5 | HP:0000548 Cone/cone-rod dystrophy; HP:0010442 Polydactyly; HP:0001249 Intellectual disability; HP:0001263 Global developmental delay; HP:0000750 Delayed speech and language development; HP:0001999 Abnormal facial shape; | Yes | BBS2  NM_031885.3:  c.[1237C>T];[1237C>T] p.[(Arg413*)]; [(Arg413*)] | Probable |  | Fauser (2003) J Med Genet 40: e104 PubMed: 12920096  Xiong (2015) Science 347: 1254806 PubMed: 25525159 |
| 38 | 176 | M | 23.3 | HP:0000510: Rod-cone dystrophy; HP:0010864 Intellectual disability, severe; HP:0000054 Micropenis; HP:0004691 2-3 toe syndactyly; HP:0000819 Diabetes mellitus; HP:0001344 Absent speech; HP:0001513 Obesity; HP:0000708 Behavioural abnormality; HP:0001250 Seizures; HP:0011003 Severe Myopia; | yes | BBS5  NM_152384.2:  c.[2T>A];[2T>A]  p.[(Met1?)];[(Met1?)] | probable | Homozygous (Mot, Fat) | Harville (2010) J Med Genet 47: 262 PubMed: 19797195 |
| 39 | 176 | F | 9.0 | HP:0000548 Cone/cone-rod dystrophy; HP:0010442 Polydactyly; HP:0001513 Obesity; HP:0001263 Global developmental delay; HP:0011003 Severe Myopia; | No, highly suspected (3 primary and 1 secondary) | BBS5  NM_152384.2:  c.[2T>A];[2T>A]  p.[(Met1?)];[(Met1?)] | probable |  | Harville (2010) J Med Genet 47: 262 PubMed: 19797195 |
| 40 | 176 | F | 2.9 | HP:0000510 Rod-cone dystrophy; HP:0100259 Postaxial polydactyly; HP:0001513 Obesity; HP:0011342 Mild global developmental delay; HP:0001156 Brachydactyly; HP:0000662 Nyctalopia; HP:0001250 Seizures | Yes | BBS7  NM_176824.2:c.[500_501insTATGAG];[500_501insTATGAG] p.[(Cys167_Gln168insMetSer)];[(Cys167_Gln168insMetSer)] | Probable | Homozygous (Mot, Fat) | Published by same patient: Taylor (2017) Ophthalmology 124: 985 PubMed: 28341476 |
| 41 | 105 | F | 8.0 | HP:0000556 Retinal dystrophy; HP:0010442 Polydactyly; HP:0001249 Intellectual disability; HP:0001513 Obesity; HP:0000750 Delayed speech and language development; HP:0000662 Nyctalopia; HP:0011003 Severe Myopia; | Yes | BBS12  NM 001178007.1:  c.[1063C>T];[1063C>T]  p.[(Arg355*)];[(Arg355*)]  (mum is het) | Probable | Homozygous (Mot, sister is homozygous) | toetzel (2007) Am J Hum Genet 80: 1 PubMed: 17160889 |
| 42 | 105 | F | 5.4 | HP:0000556: Retinal dystrophy; HP:0100259: Postaxial polydactyly; HP:0008718 Unilateral renal dysplasia; HP:0001263: Global developmental delay; HP:0001762: Talipes equinovarus; HP:0025502: Overweight; HP:0000148 Vaginal atresia; HP:0030010: Hydrometrocolpos | No, highly suspected  (3 primary and 1 secondary) | No causative mutation detected | unknown |  |  |
| 43 | 176 | M | 28.6 | HP:0000510: Rod-cone dystrophy; HP:0001513: Obesity: HP:0001118: Juvenile cataract;  HP:0001156 Brachydactyly;HP:0000648: Optic atrophy; HP:0000662: Nyctalopia; HP:0010535: Sleep apnoea; HP:0004782: Hypotrichosis of the scalp; | No, highly suspected  (2 primary and 2 secondary) | No causative mutation detected | unknown |  |  |

M=male, F=female, Mot=mother tested, Fat=Father tested

Supplemental Table S7: 1 Joubert syndrome referral.

| Pt. no. | Number of genes tested | Gender | Age at referral for testing | Phenotype (HPO terms) | Variants probably or possibly accounting  for clinical presentation | Genetic diagnosis | Segregation Analysis | Publication |
| --- | --- | --- | --- | --- | --- | --- | --- | --- |
| 44 | 105 | F | 0.6 | HP:0010442 Polydactyly;  HP:0000556 Retinal dystrophy;  HP:0001263 Global developmental delay;  HP:0001318 Muscular hypotonia  HP:0002783 Recurrent lower respiratory tract infections  NOTE: MRI head confirms Joubert suspicion | CEP290  NM_025114.3:c.[5668G>T];[5668G>T] p.[(Gly1890*)];[(Gly1890*)] | Probable | Homozygous (Mot) | Sayer (2006) Nat Genet 38: 674 PubMed: 16682973 |

F=female, Mot=mother tested

**Supplemental Table S8: Details of the 5 Senior Loken syndrome patients included in this study and corresponding genetic variants identified as probably or possibly causal of their clinical presentation by IRD panel testing.** Segregation analysis was not done on these patients.

| Pt. no. | Number of genes tested | Gender | Age at referral for testing | Phenotype (HPO terms) | Variants probably or possibly accounting  for clinical presentation | Genetic diagnosis | Publication |
| --- | --- | --- | --- | --- | --- | --- | --- |
| 45 | 176 | M | 53.9 | Referred as SLS  HP:0000083 Renal insufficiency;  HP:0007754 Macular dystrophy;  HP:0007703 Abnormality of retinal pigmentation | CEP290  NM_025114.3:c.[4723A>T];[6277del]  p.[(Lys1575*)];[(Val2093Serfs*4)] | Probable | Nonsense: Perrault (2007) Hum Mutat 28: 416 PubMed: 17345604  Frameshift deletion: Brancati (2007) Am J Hum Genet 81: 104 PubMed: 17564967 |
| 46 | 176 | F | 28.9 | Referred with FHx of SLS but does not fulfil criteria  HP:0000510 Rod-cone dystrophy | IQCB1  NM_001023570.2:c.[488-1G>A];[488-1G>A] | Probable | Otto (2008) Hum Mutat 29: 418 PubMed: 18076122  Chaki (2011) Kidney Int 80: 1239 PubMed: 21866095 |
| 47 | 176 | F | 9.3 | Referred as unknown diagnosis but fulfils diagnostic criteria for SLS  HP:0000090 Nephronophthisis;  HP:0000083 Renal insufficiency;  HP:0001510 Growth delay;  HP:0007814 Retinal pigment epithelial mottling | NPHP4  NM_015102.4:c.[2930C>T];[2029C>T]  p.[(Thr977Met)];[(Pro677Ser)] | Probable | same patient: Taylor (2017) Ophthalmology 124: 985 PubMed: 28341476 |
| 48 | 176 | M | 28.1 | Referred as unknown diagnosis but fulfils diagnostic criteria for SLS  HP:0000083: Renal insufficiency;  HP:0001146: Pigmentary retinal degeneration; HP:0007875: Congenital blindness;  HP:0000547: Tapetoretinal degeneration; HP:0006934: Congenital nystagmus | CEP290  NM_025114.3:c.[5668G>T];[5668G>T]  p.[(Gly1890*)];[(Gly1890*)] | Probable | Sayer (2006) Nat Genet 38: 674 PubMed: 16682973  Sönmez (2014) Turk J Pediatr 56: 458 PubMed: 25818971 |
| 49 | 176 | F | 58.8 | Referred as unknown diagnosis but fulfils diagnostic criteria for SLS  HP:0003774 End stage renal failure;  HP:0000510 Rod-cone dystrophy;  HP:0000518 Cataract | No mutations detected |  |  |

M=male, F=female

**Supplemental Table S9: Details of the 5 Cohen syndrome patients included in this study and corresponding genetic variants identified as probably or possibly causal of their clinical presentation by IRD panel testing.**

| Pt. no. | Number of genes tested | Gender | Age at referral for testing | Phenotype (HPO terms) | Variants probably or possibly accounting  for clinical presentation | Genetic diagnosis | Segregation Analysis | Publications |
| --- | --- | --- | --- | --- | --- | --- | --- | --- |
| 50 | 176 | F | 3.3 | HP:0001875 Neutropenia; HP:0001999 Abnormal facial shape;  HP:0007703 Abnormality of retinal pigmentation  HP:0012736 Profound global developmental delay;  HP:0000252 Microcephaly;HP:0025502 Overweight; | VPS13B  NM_017890.4:  c.[7758del];[(5983+1_5984-1)_ (7125+1_7126-1)dup]  (Exon38 to Exon42 duplication)  p.[(Lys2586Asnfs*7)];[?] | Probable | Trans (Mot) | Same patient was published by our group:  Frameshift: Taylor (2017) Ophthalmology 124: 985 PubMed: 28341476  duplication: Ellingford (2017) Eur J Hum Genet 25: 719 PubMed: 28378820 |
| 51 | 176 | M | 3.9 | HP:0000556:Retinal dystrophy; HP:0001249: Intellectual disability; HP:0001999: Abnormal facial shape;  HP:0008947: Infantile muscular hypotonia; HP:0001270 Motor delay; HP:0000252: Microcephaly; HP:0001263 Global developmental delay; HP:0008897: Postnatal growth retardation; HP:0002783: Recurrent lower respiratory tract infections; HP:0001166: Arachnodactyly; | VPS13B  NM_017890.4:  c.[6121+1G>C];[(?_-1)_(937+1_938-1)del]  (exon 1 to exon 8 deletion) | Probable | N/D | Same patient was published by our group:  Splicing: Taylor (2017) Ophthalmology 124: 985 PubMed: 28341476  deletion: Ellingford (2017) Eur J Hum Genet 25: 719 PubMed: 28378820 |
| 52 | 176 | F | 49.2 | HP:0000505: Visual Impairment;  HP:0000750: Delayed speech and language development;  HP:0000322:Short philtrum; HP:0000448:Prominent nose; HP:0001270: motor delay; HP:0000252:Microcephaly; HP:0002421: poor head control;  HP:0002019:Constipation; | VPS13B  NM_017890.4:  c.[468_471del];[10156dup]  p.[(Asn157Serfs*3)];[(Thr3386Asnfs*3)] | Probable | N/D | Frameshift deletion: novel  Frameshift duplication: Athanasakis (2012) Mol Syndromol 3: 30 PubMed: 22855652 |
| 53 | 176 | M | 20.3 | HP:0001249 Intellectual disability; HP:0000556 Retinal dystrophy; HP:0001999 Abnormal facial shape (suggestive facial features); HP:0000252 Microcephaly; HP:0001382 Joint hypermobility; HP:0000729 Autistic behaviour; HP:0002493 Upper motor neuron dysfunction; HP:0001629 Ventricular septal defect; HP:0000023 Inguinal hernia; | No pathogenic mutation detected | Unknown | N/D |  |
| 54 | 176 | M | 18.1 | HP:0001249 Intellectual disability; HP:0000556 Retinal dystrophy; HP:0001999 Abnormal facial shape (some but not all features); HP:0000252 Microcephaly; HP:0000444 Beaked nose; HP:0000322 short philtrum; HP:0000545 Myopia; | No pathogenic mutation detected | Unknown | N/D |  |

M=male, F=female, Mot=mother tested, Fat=Father tested, N/D=not done

Supplemental Table S10: Details of the single Norrie syndrome patient included in this study and corresponding genetic variant identified as probably causal of part their clinical presentation by IRD panel testing. Segregation analysis was not done on this patient.

| Pt. no. | Number of genes tested | Gender | Age at referral for testing | Phenotype (HPO terms) | Variants probably or possibly accounting for clinical presentation | Genetic diagnosis | Publication |
| --- | --- | --- | --- | --- | --- | --- | --- |
| 55 | 105 | M | 1.1 | HP:0007973:Retinal dysplasia;  HP:0000541:Retinal detachment;  (Bilateral vitreo-retinal dysplasia)  HP:0000618:Blindness;  HP:0000407:Sensorineural hearing impairment;  HP:0001250:Seizures;  HP:0000718:Aggressive behaviour | FZD4  NM_012193.3:c.[313A>G];[313=]  p.[(Met105Val)]; [(Met105=)] (cause of AD FEVR) | Probable AD FEVR | Kondo (2003) Br J Ophthalmol 87: 1291 PubMed: 14507768 |

M=male, AD FEVR =autosomal dominant Familial exudative vitreoretinopathy

Supplemental Table S11: Details of patients referred with an ‘unknown’ clinical diagnosis. Segregation analysis was not done on these patients.

| Pt. no. | Number of genes tested | Gender | Age at referral for testing | Phenotype (HPO terms) | Variants probably or possibly accounting for clinical presentation | Genetic diagnosis |
| --- | --- | --- | --- | --- | --- | --- |
| 56 | 176 | M | 0.9 | HP:0001263 Global developmental delay; HP:0000365 Hearing impairment/loss;  HP:0000821 Hypothyroidism; HP:0001508 Failure to thrive;  HP:0001410 Decreased liver function; HP:0000548 Cone/cone-rod dystrophy | MVK  NM_000431.2:c.[380C>T];[380C>T]  p.[(Pro127Leu)]; [(Pro127Leu)] | Mevalonic aciduria (AR) |
| 57 | 176 | F | 1.2 | HP:0000252 Microcephaly; HP:0011968 Feeding difficulties;  HP:0001263 Global developmental delay; HP:0007973 Retinal dysplasia;  HP:0007773 Vitreoretinopathy | KIF11  NM_004523.3:c.[478_479del];[478_479=] p.[(Leu160Valfs*5)];[( Leu160=)] | Microcephaly with or without chorioretinopathy, lymphedema, or mental retardation (AD) |
| 58 | 176 | M | 22.7 | HP:0001250 Seizures; HP:0000545 Myopia;  HP:0000510 Rod-cone dystrophy; HP:0000662 Nyctalopia;  HP:0007663 Reduced visual acuity; HP:0000518 Cataract;  HP:0000639 Nystagmus | OTX2  NM_021728.2:c.[811del];[811=]  p.[(Thr271Leufs*31)]; [(Thr271=)] | Syndromic microphthalmia type 5 (AD) |
| 59 | 176 | F | 29.6 | HP:0010978 Abnormality of immune system physiology; HP:0000510 Rod-cone dystrophy; | MVK  NM_000431.2:c.[630G>A];[1129G>A]  p.[(Trp210*)];[(Val377Ile)] | Hyper-IgD syndrome (AR) |
| 60 | 176 | F | 50.9 | HP:0001251 Cerebellar ataxia;  HP:0000510 Rod-cone dystrophy; | IFT140  NM_014714.3:c.[998G>A];[998G>A]  p.[(Cys333Tyr)];[(Cys333Tyr)] | Short-Rib Thoracic Dysplasia 9 With Or Without Polydactyly |
| 61 | 176 | M | 8.5 | HP:0000662 Nyctalopia; HP:0000510: Rod-cone dystrophy;  HP:0001249 Intellectual disability | BBS1  NM_024649.4:c.[1110G>A];[1110G>A]  p.[(Pro370Pro)];[(Pro370Pro)] | Bardet-Biedl syndrome type 1 |
| 62 | 176 | F | 36.2 | HP:0000510 Rod-cone dystrophy; HP:0010442 Polydactyly;  HP:0002099 Asthma; HP:0100502 Vitamin B12 deficiency | BBS1  NM_024649.4:c.[1169T>G];[1169T>G]  p.[(Met390Arg)];[(Met390Arg)] | Bardet-Biedl syndrome type 1 |
| 63 | 176 | M | 14.2 | HP:0001249 Intellectual disability;  HP:0000708 Behavioural abnormality;  HP:0007642 Congenital stationary night blindness | *TRPM1*  NM_002420.5:c.[2951G>A] p.[(Arg984His)] Heterozygous;  (microarray identified a 15q13.3 microdeletion reported in the loss of the TRPM1 gene:  NM_002420.5:c.(?_−1)_(*1_?)del) | Congenital Stationary Night Blindness  (Possible diagnosis by panel) |
| 64 | 176 | M | 4.9 | HP:0000750 Delayed speech and language development;  HP:0000548 Cone/cone-rod dystrophy | GNAT2  NM_005272.3:c.[605G>A];[605G>A]  p.[(Gly202Glu)];[(Gly202Glu)] | Autosomal Recessive Cone dystrophy |
| 65 | 176 | M | 6.9 | HP:0001249 Intellectual disability;  HP:0000548 Cone/cone-rod dystrophy | CNGA3  NM_001298.2:c.[560T>C];[560T>C]  p.[(Ile187Thr)];[(Ile187Thr)] | Autosomal Recessive Cone dystrophy |
| 66 | 176 | F | 16.5 | HP:0001251 Ataxia;  HP:0000556 Retinal dystrophy | PROM1  NM_006017.2:c.[1354dup];[1354dup]  p.[(Tyr452Leufs*13)];[(Tyr452Leufs*13)] | Autosomal Recessive Retinal Dystrophy |
| 67 | 105 | M | 33.6 | HP:0002652 Skeletal dysplasia;  HP:0000510 Rod-cone dystrophy | PRPF3  NM_004698.2:c.[1285G>T]; [1285=]  p.[(Asp429Tyr)];[(Asp429=)] | Autosomal Dominant Retinal Dystrophy |
| 68 | 176 | M | 37.2 | HP:0001513 Obesity;  HP:0000510 Rod-cone dystrophy; | EYS  NM_001142800.1:c.[8133_8137del]; [8816G>C]  p.[(Phe2712Cysfs*33)]; [(Cys2939Ser)] | Autosomal Recessive Retinal Dystrophy |
| 69 | 105 | M | 49.2 | HP:0001249 Intellectual disability;  HP:0000510 Rod-cone dystrophy | USH2A  NM_206933.2:c.[6670G>T];[10342G>A]  p.[(Gly2224Cys)];[(Glu3448Lys)] | Autosomal Recessive Retinal Dystrophy |
| 70 | 176 | M | 0.0 | HP:0001789 Hydrops fetalis;  HP:0001319 Neonatal hypotonia;  HP:0001263 Global developmental delay;  HP:0000541 Retinal detachment;  HP:0007973 Retinal dysplasia | No pathogenic mutation detected.  Research WES found a de novo heterozygous mutation in *PRKAR1A*  NM_002734.4:c.[1102C>T];[1102=] p.[(Arg368*)];[(Arg368=)] | Autosomal Dominant Acrodysostosis type1 |
| 71 | 176 | M | 2.6 | HP:0001263 Global developmental delay; HP:0001004 Lymphoedema;  HP:0000252 Microcephaly; HP:0000543 Optic disc pallor | No pathogenic mutation detected | Unknown |
| 72 | 176 | F | 13.7 | HP:0002194 Delayed gross motor development; HP:0007370: Aplasia/Hypoplasia of the corpus callosum; HP:0007957 Corneal opacity;  HP:0000556 Retinal dystrophy | No pathogenic mutation detected | Unknown |
| 73 | 176 | F | 7.8 | HP:0025502 Overweight; HP:0000510 Rod-cone dystrophy;  HP:0000750 Delayed speech and language development; | No pathogenic mutation detected | Unknown |
| 74 | 105 | M | 9.8 | HP:0004464 Postauricular pits; HP:0430015 Abnormality of pharyngeal musculature; HP:0000316 Hypertelorism; HP:0000488 Retinopathy;  HP:0000177: Abnormality of upper lip | No pathogenic mutation detected | Unknown |
| 75 | 105 | F | 12.4 | HP:0001249 Intellectual disability;  HP:0000510 Rod-cone dystrophy; | No pathogenic mutation detected | Unknown |
| 76 | 177 | F | 4.1 | HP:0000252 Microcephaly;  HP:0007773 Vitreoretinopathy | No pathogenic mutation detected | Unknown |
| 77 | 176 | F | 4.3 | HP:0001263 Global developmental delay; HP:0001272 Cerebellar atrophy; HP:0000556 Retinal dystrophy | No pathogenic mutation detected | Unknown |
| 78 | 105 | F | 18.6 | HP:0000365 Hearing impairment/loss; HP:0007754 Macular dystrophy | No pathogenic mutation detected | Unknown |
| 79 | 176 | M | 27.4 | HP:0030984 Abnormal serum bile acid concentration; HP:0000164 Abnormality of the dentition; HP:0000510 Rod-cone dystrophy | No pathogenic mutation detected | Unknown |
| 80 | 175 | M | 0.5 | HP:0100540 Palpebral edema; HP:0000252 Microcephaly;  HP:0000532 Chorioretinal abnormality | No pathogenic mutation detected | Unknown |
| 81 | 176 | M | 1.5 | HP:0001263 Global developmental delay; HP:0000556 Retinal dystrophy | No pathogenic mutation detected | Unknown |
| 82 | 176 | F | 2.3 | HP:0000365 Hearing impairment;HP:0000639 Nystagmus;  HP:0007401 Macular atrophy | No pathogenic mutation detected | Unknown |
| 83 | 105 | F | 12.3 | HP:0004322 Short stature; HP:0000639 Nystagmus;  HP:0000548 Cone/cone-rod dystrophy; | No pathogenic mutation detected | Unknown |
| 84 | 176 | M | 13.9 | HP:0001249 Intellectual disability; HP:0011003 Severe Myopia; HP:0007703 Abnormality of retinal pigmentation | No pathogenic mutation detected | Unknown |
| 85 | 105 | F | 15.5 | HP:0000729 Autism spectrum disorder; HP:0000510 Rod-cone dystrophy | No pathogenic mutation detected | Unknown |
| 86 | 105 | F | 18.5 | HP:0001730 Progressive hearing impairment; HP:0000548 Cone rod dystrophy | No pathogenic mutation detected | Unknown |
| 87 | 105 | M | 28.8 | HP:0000408 Progressive sensorineural hearing impairment; HP:0008615 Adult onset sensorineural hearing impairment; HP:0000556: Retinal dystrophy; HP:0007830: Adult-onset night blindness; HP:0007663: Reduced visual acuity; HP:0000519: Congenital cataract | No pathogenic mutation detected | Unknown |
| 88 | 105 | F | 38.3 | HP:0001251 Ataxia; HP:0000510 Rod-cone dystrophy | No pathogenic mutation detected | Unknown |
| 89 | 105 | F | 58.9 | HP:0000365 Hearing impairment; HP:0000510 Rod-cone dystrophy | No pathogenic mutation detected | Unknown |
| 90 | 105 | M | 46.7 | HP:0000365 Hearing impairment; HP:0000556 Retinal dystrophy | No pathogenic mutation detected | Unknown |
| 91 | 176 | F | 7.5 | Foetal alcohol syndrome (HP:0001263 Global developmental delay; HP:0001249 Intellectual disability; HP:0001510 Growth delay; HP:0001999 Abnormal facial shape; HP:0000252 Microcephaly) ; HP:0000505 Visual impairment (ERG features of retinal dystrophy but not typical of RP, high hypermetropia, optic nerve hypoplasia, FHx of ADRP); HP:0005681 Juvenile rheumatoid arthritis | No pathogenic mutation detected | Unknown |
| 92 | 105 | M | 0.7 | HP:0008936 Muscular hypotonia of the trunk; HP:0001263 Global developmental delay;  HP:0000556 Retinal dystrophy | No pathogenic mutation detected | Unknown |
| 93 | 176 | F | 9.2 | HP:0001956 Truncal obesity;  HP:0009128 Aplasia/Hypoplasia involving the musculature of the extremities;  HP:0000556 Retinal dystrophy | No pathogenic mutation detected | Unknown |
| 94 | 176 | M | 10.0 | HP:0001249 Intellectual disability; HP:0001999 Abnormal facial shape; HP:0000556 Retinal dystrophy | No pathogenic mutation detected | Unknown |
| 95 | 105 | M | 15.6 | HP:0010442 Polydactyly; HP:0002783 Recurrent chest infections; HP:0000556 Retinal dystrophy | No pathogenic mutation detected | Unknown |
| 96 | 105 | M | 25.2 | HP:0001249 Intellectual disability; HP:0001650 Aortic valve stenosis; HP:0000662 Nyctalopia; HP:0007994 Peripheral visual field loss;  HP:0000510 Rod-cone dystrophy; HP:0000666 Horizontal nystagmus | No pathogenic mutation detected | Unknown |
| 97 | 176 | F | 46.9 | HP:0000365 Hearing impairment/loss; HP:0002758 Osteoarthritis;  HP:0000662 Nyctalopia; HP:0000548 Cone/cone-rod dystrophy | No pathogenic mutation detected | Unknown |
| 98 | 105 | F | 53.6 | HP:0012715 Profound hearing impairment; HP:0003774 End stage renal failure; HP:0000556 Retinal dystrophy | No pathogenic mutation detected | Unknown |
| 99 | 105 | M | 55.4 | HP:0001251 Ataxia; HP:0001305 Dandy-Walker malformation;  HP:0000556 Retinal dystrophy | No pathogenic mutation detected | Unknown |
| 100 | 176 | M | 69.5 | HP:0000819 Diabetes mellitus; HP:0000365 Hearing impairment;  HP:0007754 Macular dystrophy | No pathogenic mutation detected | Unknown |
| 101 | 105 | M | 10.3 | HP:0004324 Increased body weight; HP:0000708 Behavioral abnormality; HP:0000548 Cone/cone-rod dystrophy | No pathogenic mutation detected | Unknown |
| 102 | 105 | M | 13.3 | HP:0001270 Motor delay; HP:0001249 Intellectual disability;  HP:0000717 Autism; HP:0100716 Self-injurious behaviour;  HP:0000556 Retinal dystrophy | No pathogenic mutation detected | Unknown |
| 103 | 176 | F | 22.7 | HP:0002019: Constipation; HP:0000010:Recurrent urinary tract infections; HP:0500001: Body odour;  HP:0025502: Overweight;  HP:0000510 Rod-cone dystrophy; | No pathogenic mutation detected | Unknown |
| 104 | 176 | M | 38.1 | HP:0000105 Enlarged kidney; HP:0000822 Hypertension; HP:0001635 Congestive heart failure; HP:0002401 Stroke-like episode; HP:0000519 Congenital cataract; HP:0000556 Retinal dystrophy | No pathogenic mutation detected | Unknown |
| 105 | 105 | F | 8.0 | HP:0000407 Hearing loss, sensorineural; HP:0000280: Coarse facial features; HP:0001357: Plagiocephaly; HP:0002942:Thoracic kyphosis;  HP:0001433: Hepatosplenomegaly; HP:0001263: Global developmental delay; HP:0000488 Retinopathy; | No pathogenic mutation detected | Unknown |
| 106 | 105 | F | 25.8 | HP:0010864 Intellectual disability, severe; HP:0000939 Osteoporosis;  HP:0001263 Global developmental delay; HP:0001250 Seizures;  HP:0000365 Hearing impairment; HP:0001956 Truncal obesity;  HP:0001751 Vestibular dysfunction; HP:0000548 Cone/cone-rod dystrophy | No pathogenic mutation detected | Unknown |
| M=male, F=female, AD=autosomal dominant, AR= autosomal recessive | | | | | | |
