# Supplementary material for: Diagnostic yield of panel-based genetic testing in syndromic inherited retinal disease
Source: Eur J Hum Genet. 2019 Dec 13;28(5):576–86. doi: 10.1038/s41431-019-0548-5 (PMC7171123; doi:10.1038/s41431-019-0548-5)
Supplement: Supplementary file 2 — Supplemental_figures_SyndromicIRD_supp-Figure-S1-S3 [file 41431_2019_548_MOESM2_ESM.docx]

**SUPPLEMENTAL MATERIAL (FIGURES)**


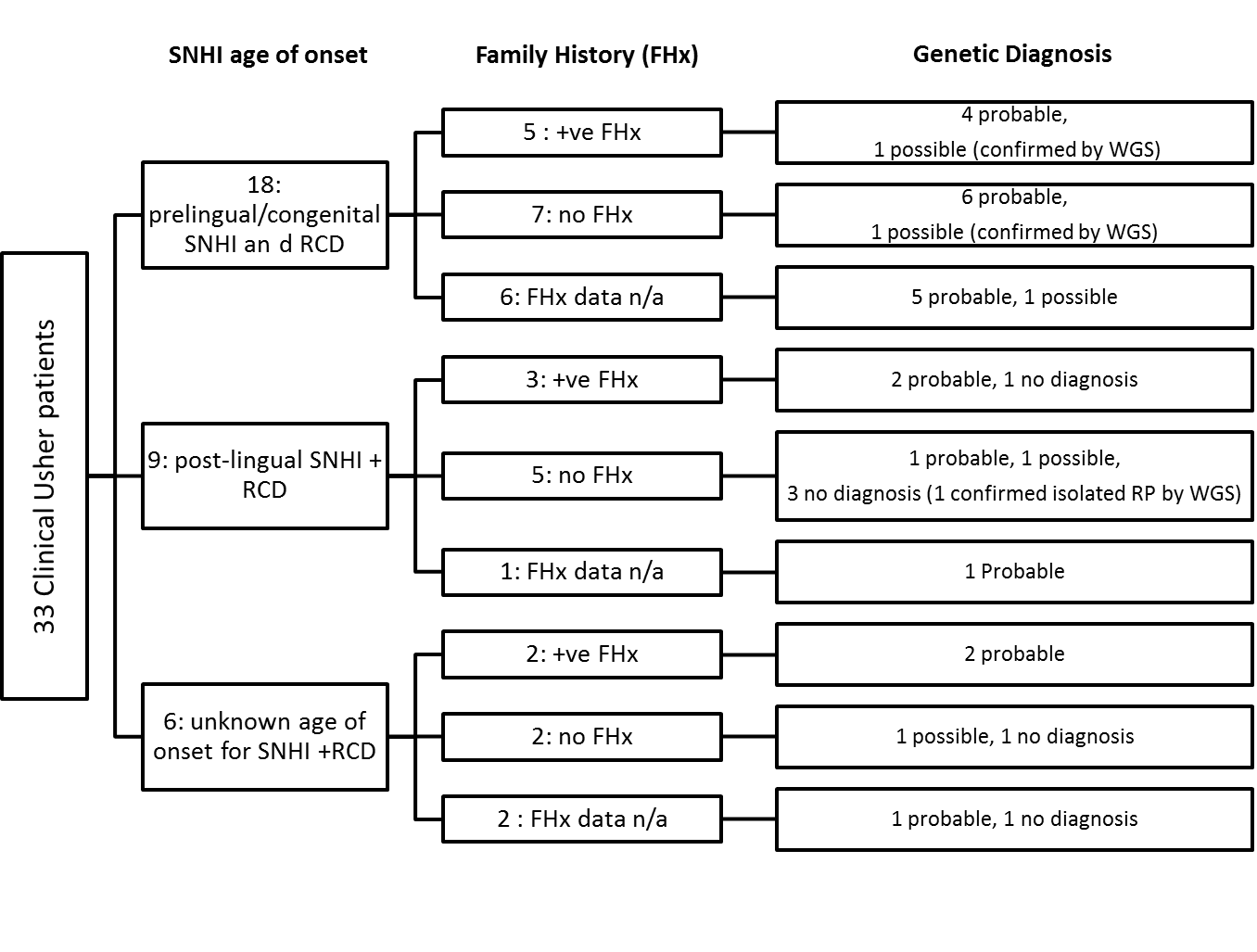


Supplemental figure S1: Flowchart of the classification of 33 patients with a provisional clinical diagnosis of Usher syndrome. SNHI= sensorineural hearing impairment, RCD= rod-cone dystrophy, WGS= whole genome sequencing, Dx=diagnosis, n/a=not available.


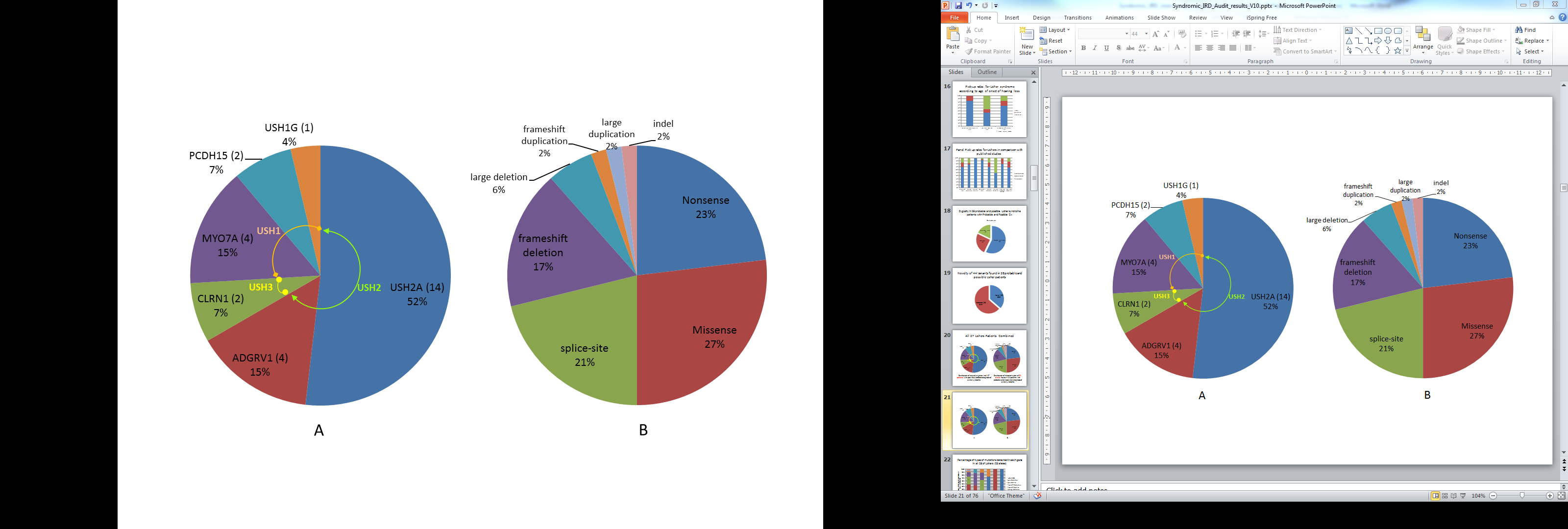


**Supplemental figure S2:** Gene mutation rates and variant types in 27 genetically diagnosed Usher syndrome patients. (A) Distribution of causative genes in all 27 patients with possible/probable diagnosis of Usher syndrome. The most commonly mutated gene was *USH2A* (14 patients), followed by *ADGRV1*(4 patients), *MYO7A* (4 patients), *PCDH15*, and *CLRN1* (2 each) and *USH1G* in 1 patient; (B) Distribution of variant types across 51 alleles appraised as ‘probably’ or ‘possibly’ disease-associated found in 27 patients with a clinical diagnosis of Usher syndrome. The most common type of variant was missense found in 27% (n=14), followed by nonsense (22%, n=12)


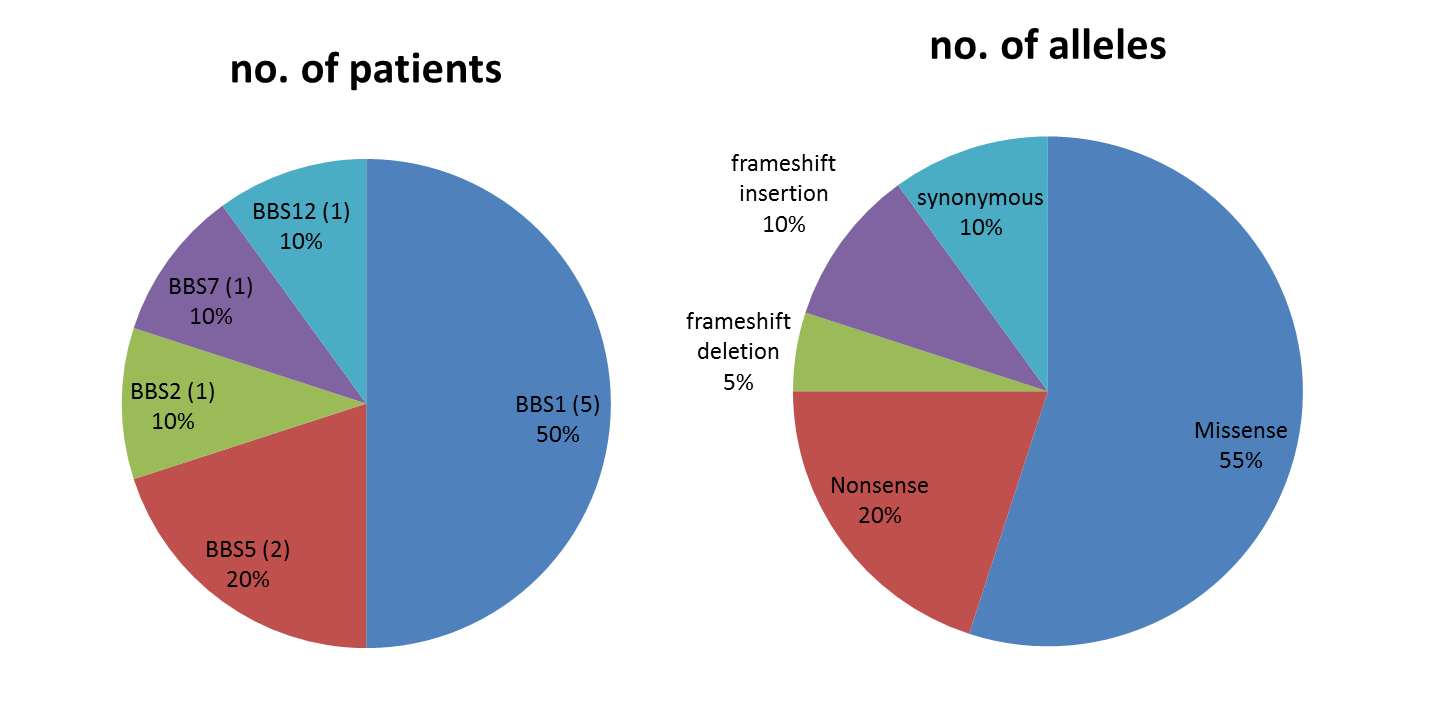


**B**

**A**

**Supplemental figure S3:** Genes and types of variants found in 10 genetically diagnosed BBS patients. (A) Variants in *BBS1* were causative in 5 (50%) patients, *BBS5* in 2 patients (20%), and variants in *BBS2, BBS7* and *BBS12* in one patient each. (B) Distribution of variant types found in 20 alleles. The most common type of variant was missense, found in 11 alleles. This was followed by nonsense (4 alleles), frameshift insertion (2), synonymous (2) and frameshift deletion (1).
